# Yinchenhao Decoction Alleviates Liver Fibrosis by Regulating Bile Acid Metabolism and TGF-β/Smad/ERK Signalling Pathway

**DOI:** 10.1038/s41598-018-33669-4

**Published:** 2018-10-18

**Authors:** Fei-Fei Cai, Rong Wu, Ya-Nan Song, Ai-Zhen Xiong, Xiao-Le Chen, Meng-Die Yang, Li Yang, Yuanjia Hu, Ming-Yu Sun, Shi-Bing Su

**Affiliations:** 1 Research Center for Traditional Chinese Medicine Complexity System, Shanghai University of Traditional Chinese Medicine, Shanghai, China; 2 The MOE Key Laboratory for Standardization of Chinese Medicines, Institute of Chinese Materia Medica, Shanghai University of Traditional Chinese Medicine, Shanghai, China; 3 State Key Laboratory of Quality Research in Chinese Medicine Institute of Chinese Medical Sciences, University of Macau, Macao, SAR China; 4 Liver disease institute, Shuguang Hospital, Shanghai University of Traditional Chinese Medicine, Shanghai, China

## Abstract

Yinchenhao decoction (YCHD), comprising Yinchenhao (*Artemisiae Scopariae* Herba), Zhizi (*Gardeniae Fructus*) and Dahuang (*Radix Rhei et Rhizoma*), is widely used for treating various diseases. We aimed to investigate the bile acid metabolic mechanism of YCHD in dimethylnitrosamine (DMN)-induced liver fibrosis model. Rats received DMN (10 mg/kg, intraperitoneally) for four successive weeks for liver fibrosis induction and were treated with YCHD for the last 2 weeks. Histopathological analysis showed that YCHD prevented DMN-induced histopathological changes in liver tissues. Serum liver function in YCHD group improved. Ultraperformance liquid chromatography-mass spectrometry analysis showed that YCHD significantly restored both free and conjugated bile acid levels increased by DMN, to normal levels. RT-qPCR results showed that YCHD treatment upregulated the expression of genes related to bile acid synthesis, reabsorption, and excretion. Western blotting analysis showed that YCHD downregulated α-SMA, TGF-β1, p-Smad3, and p-ERK1/2 expression in chenodeoxycholic acid (CDCA)-activated hepatic stellate cells (HSCs). The viability of CDCA-activated HSCs significantly increased after treatment with YCHD and PD98059 (an ERK inhibitor) compared to YCHD treatment alone. Our findings suggest that YCHD alleviated DMN-induced liver fibrosis by regulating enzymes responsible for bile acid metabolism. Additionally, it inhibits CDCA-induced HSC proliferation and activation via TGF-β1/Smad/ERK signalling pathway.

## Introduction

Liver fibrosis is a reversible wound-healing response to hepatocyte injury caused by various acute or chronic liver diseases. Fibrosis is characterised by the excessive synthesis and deposition of extracellular matrix (ECM) in various tissues^1–3^. In case of acute and self-limiting liver injury, ECM hyperplasia is temporary, after which the liver returns to normal. However, if this damage persists, it will result in chronic inflammation, excessive ECM production, and replacement of parenchymal cells with scar tissue cells^4^. HSCs are regarded as the primary effector of hepatic fibrosis because they participate in ECM deposition and triggers an immune response through the secretion of cytokines and chemokines^5^. Therefore, fibrosis may be countered by inhibiting the proliferation and activation of HSCs.

Bile acids are steroid acids found predominantly in the bile of mammals and other vertebrates. Cholestatic liver diseases are characterised by the dysfunction of bile acid metabolism, including bile acid synthesis, reabsorption, and excretion. The accumulation of bile acids in the liver induces hepatocyte apoptosis and liver injury, thereby causing hepatic fibrosis^6^. Primary bile acids (hydrophobic), also known as toxic bile acids, are involved in cholestatic liver injury^7^. Chenodeoxycholic acid (CDCA), one of the major hydrophobic primary bile acids, induces HSC activation and hepatic fibrosis^8^.

Traditional Chinese medicine (TCM) has been used from ancient times for the treatment of various diseases^9–12^. As a classic traditional Chinese formula widely used for treating various liver diseases, Yinchenhao decoction (YCHD) was first described in the Treatise on Febrile Diseases (Shanghan Lun). The components of YCHD, Yinchenhao, Zhizi and Dahuang, have been widely used for treating diseases such a liver and kidney diseases^13–17^. YCHD improves biochemical and histological statuses in nonalcoholic fatty liver disease^18^ and protects against dimethylnitrosamine (DMN)-induced inflammatory injury in hepatic parenchymal cells in rats^19^. However, the mechanisms underlying the hepatoprotective effects of YCHD remain to be elucidated.

In this study, we used a DMN-induced chronic liver injury rat model to investigate the mechanisms of YCHD against liver fibrosis and the underlying metabolic profiles of bile acids. Moreover, we investigated whether YCHD inhibits CDCA-induced HSC activation through TGF-β1/Smad/ERK signalling pathway.

## Results

### YCHD Attenuates DMN-induced Hepatic Histopathological Changes

**Figure 1:**
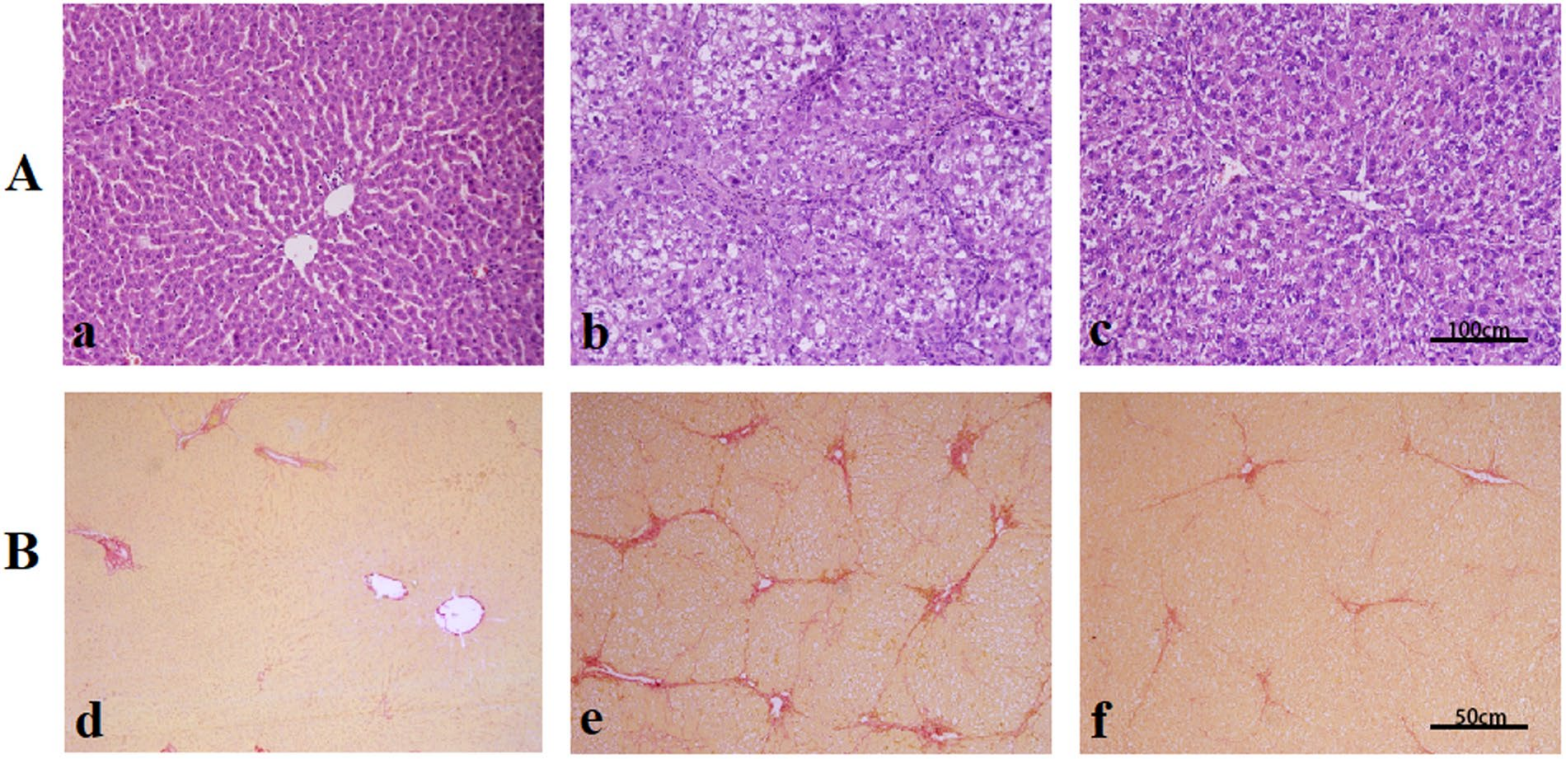
Effects of YCHD on histological changes of liver. (**A**) Hematoxylin and eosin (H&E) staining ×200; (a) control group; (b) DMN-induced group; (c) YCHD-treated group. (**B**) Sirius red staining ×100; (d) control group; (e) DMN-induced group; (f) YCHD-treated group.

Haematoxylin and eosin (H&E) staining showed that hepatocytes in rats receiving DMN alone exhibited increased swelling, degeneration, and lymphocyte infiltration in intralobular, periportal, and bridging areas. Treatment with YCHD clearly improved DMN-induced pathological changes. No remarkable hepatocyte degeneration was found in the liver tissues of YCHD-treated rats (Fig. 1A).

In the control group, Sirius red staining showed a normal distribution of collagen with a thin rim around the terminal hepatic vein. Liver specimens obtained from DMN-treated rats showed extensive perilobular fibrosis as indicated by increased hepatic collagen in comparison with control rats. YCHD-treated rats showed decreased collagen deposition (Fig. 1B). Significantly higher fibrosis scores were observed in DMN-treated rats than in normal control rats; YCHD significantly improved these scores compared with DMN (Table 1).

**Table 1:** Effects of YCHD on liver hydroxyproline (Hyp) content and fibrotic grade.

Groups	Hyp content (µg/g wet liver)	Fibrotic grade				
		0	I	II	III	IV
Control (n = 9)	181.78 ± 43.45	9	0	0	0	0
DMN (n = 9)	427.90 ± 129.46^##^	0	0	1	5	3
YCHD (n = 6)	204.39 ± 48.11**	0	0	6	0	0

grade 0: normal; grade I: very slight; grade II: slight; grade III: moderate; grade IV: severe. Data are expressed as numbers of animals with each fibrotic grade. ^##^P < 0.01 (vs. Control); ^**^P < 0.01 (vs. DMN).

### YCHD Reduces Hepatic hydroxyproline (Hyp) Content and Improves Serum Liver Function

**Figure 2:**
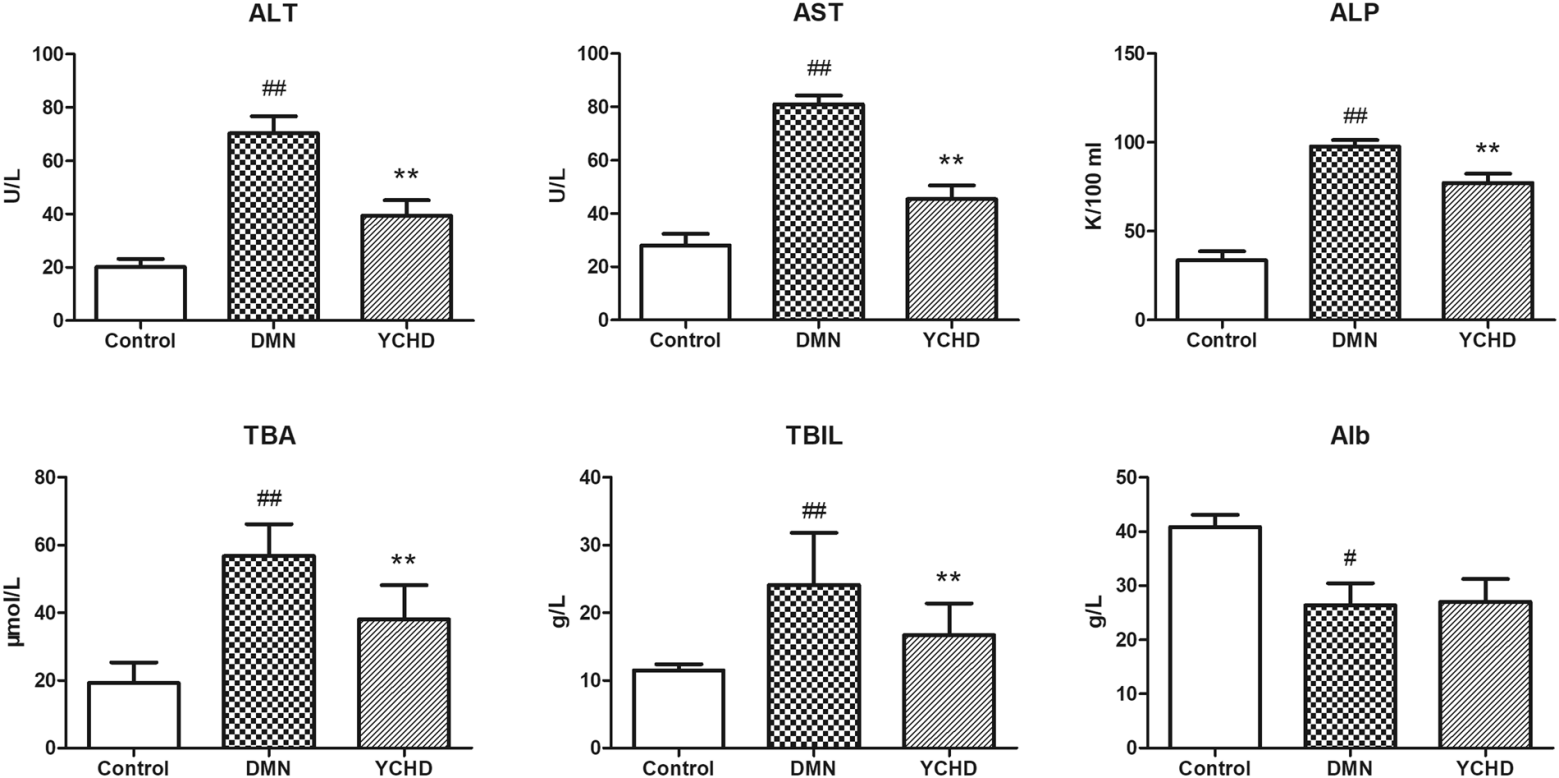
Effects of YCHD on serum activities of AST, ALT, and ALP and serum content of TBA and TBIL in normal control, DMN, and YCHD groups. ^#^*P* < 0.05 (vs. Control), ^##^*P* < 0.01 (vs. Control), **P < 0.01 (vs. DMN).

To evaluate the anti-fibrosis effects of YCHD further, Hyp content in liver tissues was measured. Compared with the DMN group, the YCHD group showed a significant decrease in Hyp content (Table 1). Rats with DMN-induced liver damage exhibited significantly increased serum alanine aminotransferase (ALT), aspartate aminotransferase (AST), and alkaline phosphatase (ALP) activities (*P* < 0.01), and serum contents of total bile acid (TBA) and total bilirubin (TBIL) (*P* < 0.01), along with reduced serum albumin (Alb) level when compared with control rats. YCHD treatment completely reversed these indices of DMN-induced damage in liver function (Fig. 2).

### YCHD Regulates Bile Acid Metabolism

**Figure 3:**
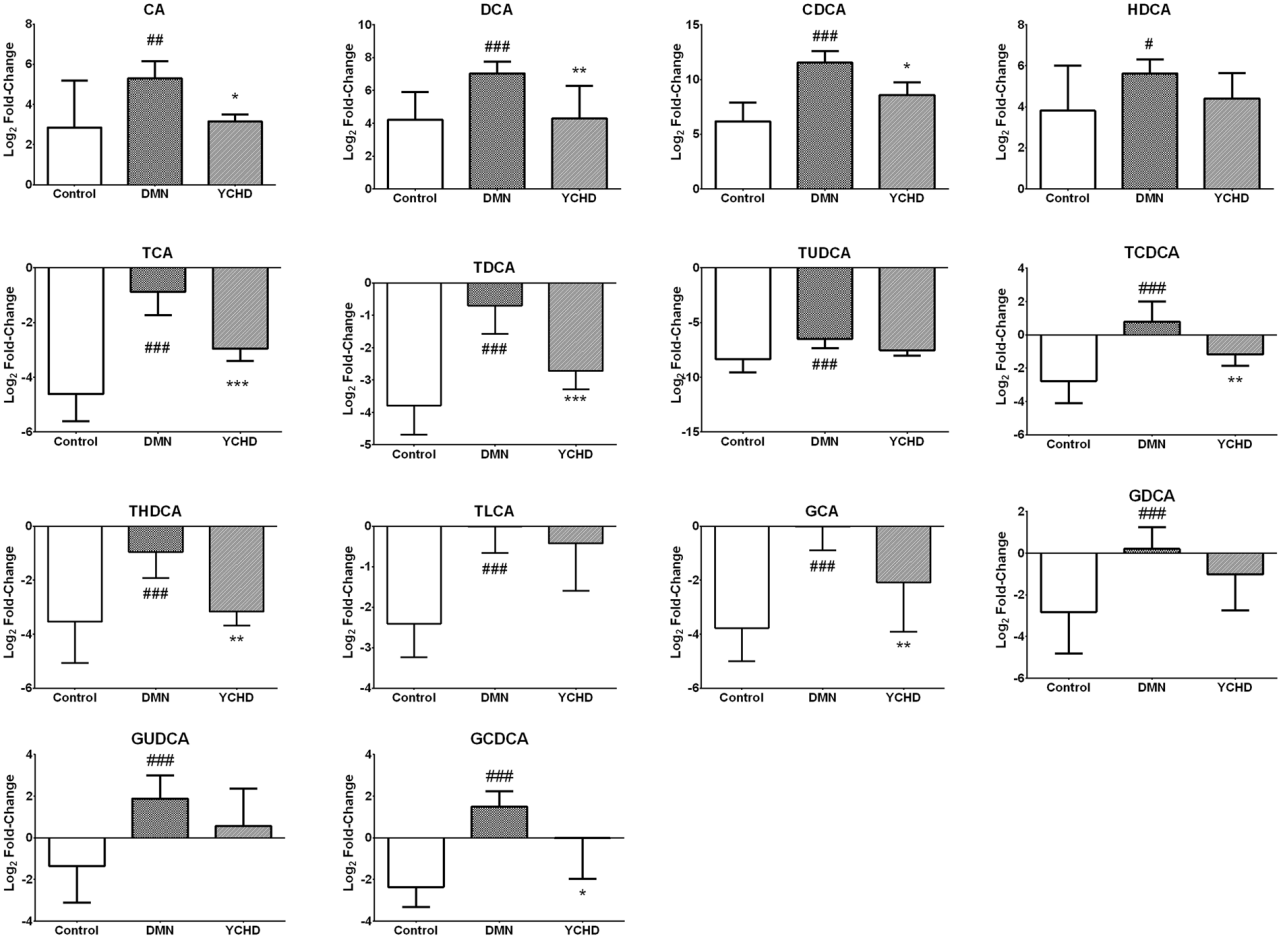
Changes of bile acids treated by YCHD. Bile acids with log_2_ fold change **>**0 or <0 were designated as increased or decreased serum bile acids compared with bile acids in hepatic tissue, respectively. All data were generated through UPLC/MS analysis. Values are expressed as mean ± SD. Significant differences were based on one-way ANOVA analysis.

For the metabolic profiling of bile acids after YCHD treatment, 16 individual bile acids in the serum and hepatic tissues (see Supplementary Figs S1 and S2) of rats were quantified using ultra performance liquid chromatography-mass spectrometry (UPLC-MS). Six free bile acids including cholic acid (CA), ursodeoxycholic acid (UDCA), CDCA, hyodeoxycholic acid (HDCA), and lithocholic acid (LCA), six taurine-conjugated bile acids including taurocholate (TCA), taurodeoxycholate (TDCA), tauroursodeoxycholate (TUDCA), taurochenodeoxycholate (TCDCA), taurohyodeoxycholic acid (THDCA), and taurolithocholicacid (TLCA), and four glycine-conjugated bile acids including glycocholate (GCA), glycodeoxycholate (GDCA), glycoursodeoxycholate (GUDCA), and glycochenodeoxycholate (GCDCA) were quantified. The changes in bile acid composition after YCHD treatment were identified by comparing the relative bile acid contents in the serum and hepatic tissues of rats (Fig. 3). UDCA and LCA contents in the liver were lower than the limit of quantitation. The levels of all other free, taurine-conjugated, and glycine-conjugated bile acids were significantly upregulated in the DMN-treated rat samples compared with control samples; however, these levels recovered to normal values upon YCHD treatment.

### YCHD Regulates the Expression of Bile Acid Metabolism-Associated Genes

**Figure 4:**
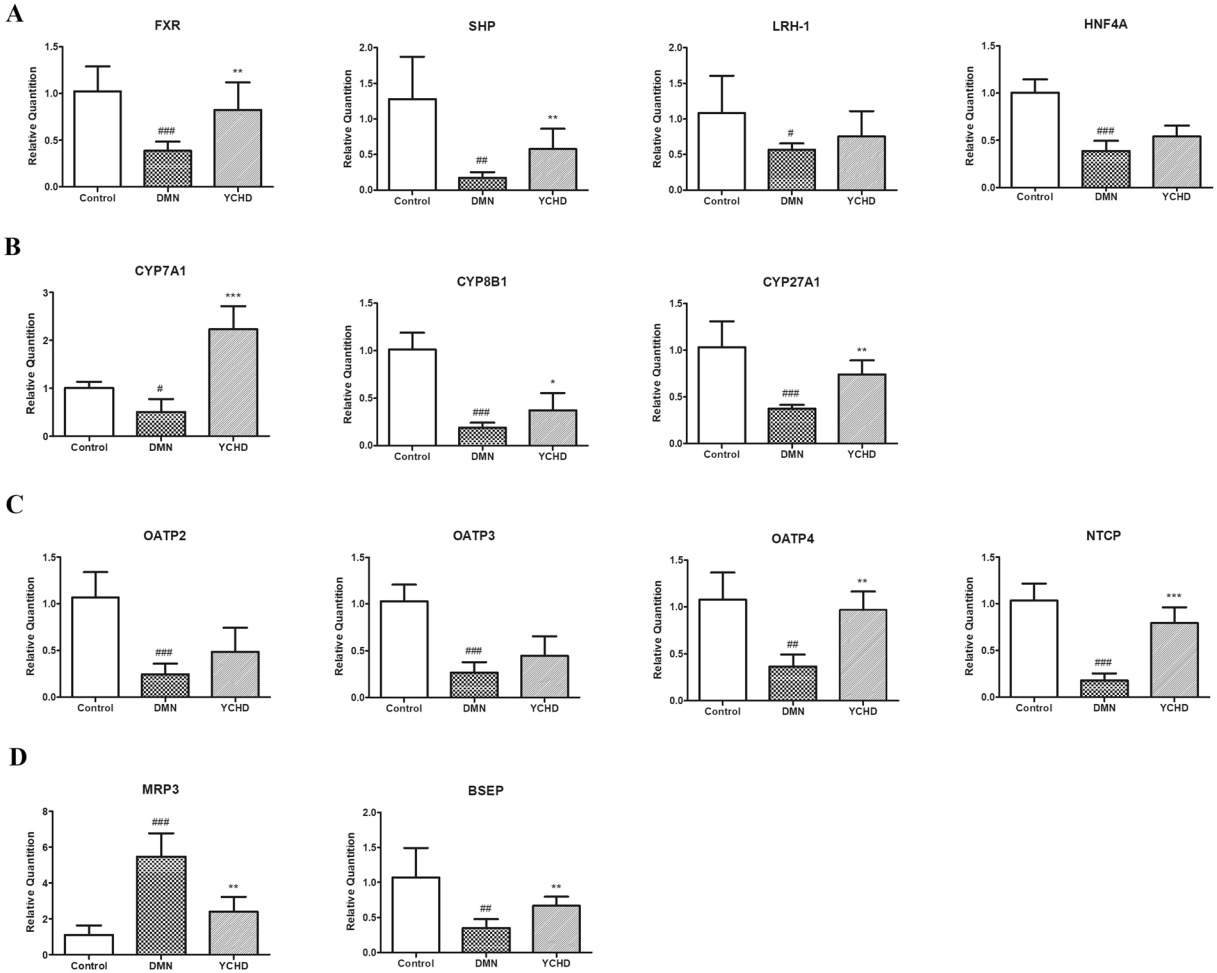
Relative expression of bile acid metabolism associated genes. (**A**) Bile acid metabolism associated nuclear receptor genes. (**B**) Bile acid synthesis associated genes. (**C**) Bile acid reabsorption associated genes. (**D**) Bile acid excretion associated genes. All data were determined by RT-qPCR. ACTB was as the internal standard. Values are expressed as mean ± SD. Significant differences were analyzed by one-way ANOVA. ^#^*P* < 0.05, ^##^*P* < 0.01, ^###^*P* < 0.0001 (vs. Control group).

To clarify the regulatory mechanisms of YCHD on bile acid metabolism, we further quantified the expression of bile acid metabolism-associated genes in rat liver tissues. The expression of nuclear receptor genes, including farnesoid X receptor (FXR), small heterodimer partner (SHP), liver receptor homolog-1 (LRH-1), and hepatocyte nuclear factor-4α (HNF4α), was markedly downregulated in the DMN group; however, FXR and SHP expression was significantly upregulated upon YCHD treatment (Fig. 4A). The expression of bile acid synthesis-associated genes, including cholesterol 7-alpha-hydroxylase (CYP7A1), sterol 12-alpha-hydroxylase (CYP8B1), and sterol 27-hydroxylase (CYP27A1), was downregulated in the DMN group compared with the control group, and YCHD treatment restored their expression to normal levels (Fig. 4B). Although the expression of bile acid reabsorption-associated genes, including organic anion-transporting polypeptides (OATP) 2, 3, and 4, and Na+-taurocholate co-transporting protein (NTCP), was downregulated in the DMN group compared with the control group, the expression of OATP4 and NTCP was significantly upregulated in the YCHD group compared with the DMN group (Fig. 4C). Moreover, the expression of bile acid excretion-associated genes, including multidrug-resistance-associated protein 3 (MRP3) and bile salt export pump (BSEP), was studied. MRP3 expression was significantly upregulated in the DMN group and significantly downregulated in the YCHD group. The results for BSEP expression were the opposite of those seen for MRP3 (Fig. 4D).

### YCHD Inhibits the Expression of TGF-β1, p-Smad3, and p-ERK1/2 in Hepatic Tissues of Rats

**Figure 5:**
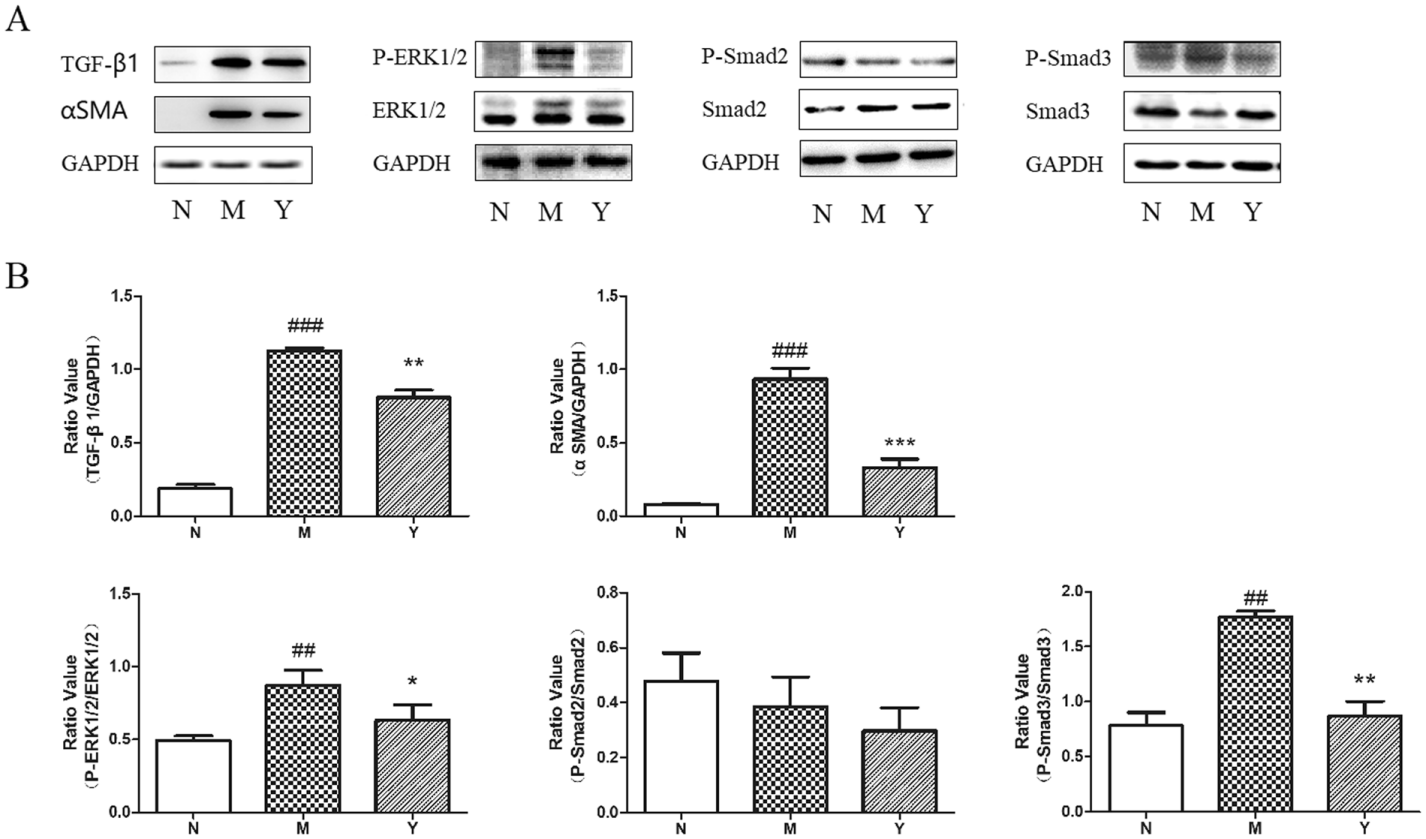
Effect of YCHD on TGF-β1, α-SMA, p-Smad2/3, and p-ERK1/2 levels in DMN-induced rats. (**A**) Western blotting image for the expression of TGF-β1, α-SMA, p-Smad2, p-Smad3, and p-ERK1/2 in normal group (N), DMN group (M), and YCHD group (Y). (**B**) The histograms displayed relative abundance, represented by the band intensity in western blotting.

Western blot analysis revealed that TGFβ1, α-SMA, p-Smad3, and p-ERK1/2 protein expression levels in DMN-induced liver fibrosis group were significantly higher than those in the control group (Fig. 5). Furthermore, YCHD treatment significantly reduced these protein expression levels. However, there was no significant difference among the DMN, control, and YCHD groups in the protein expression levels of p-Smad2.

### YCHD Suppresses CDCA-induced HSC-T6 Cell Proliferation and Activation by Inhibiting TGF-β1/Smad/ERK Signalling Pathways

**Figure 6:**
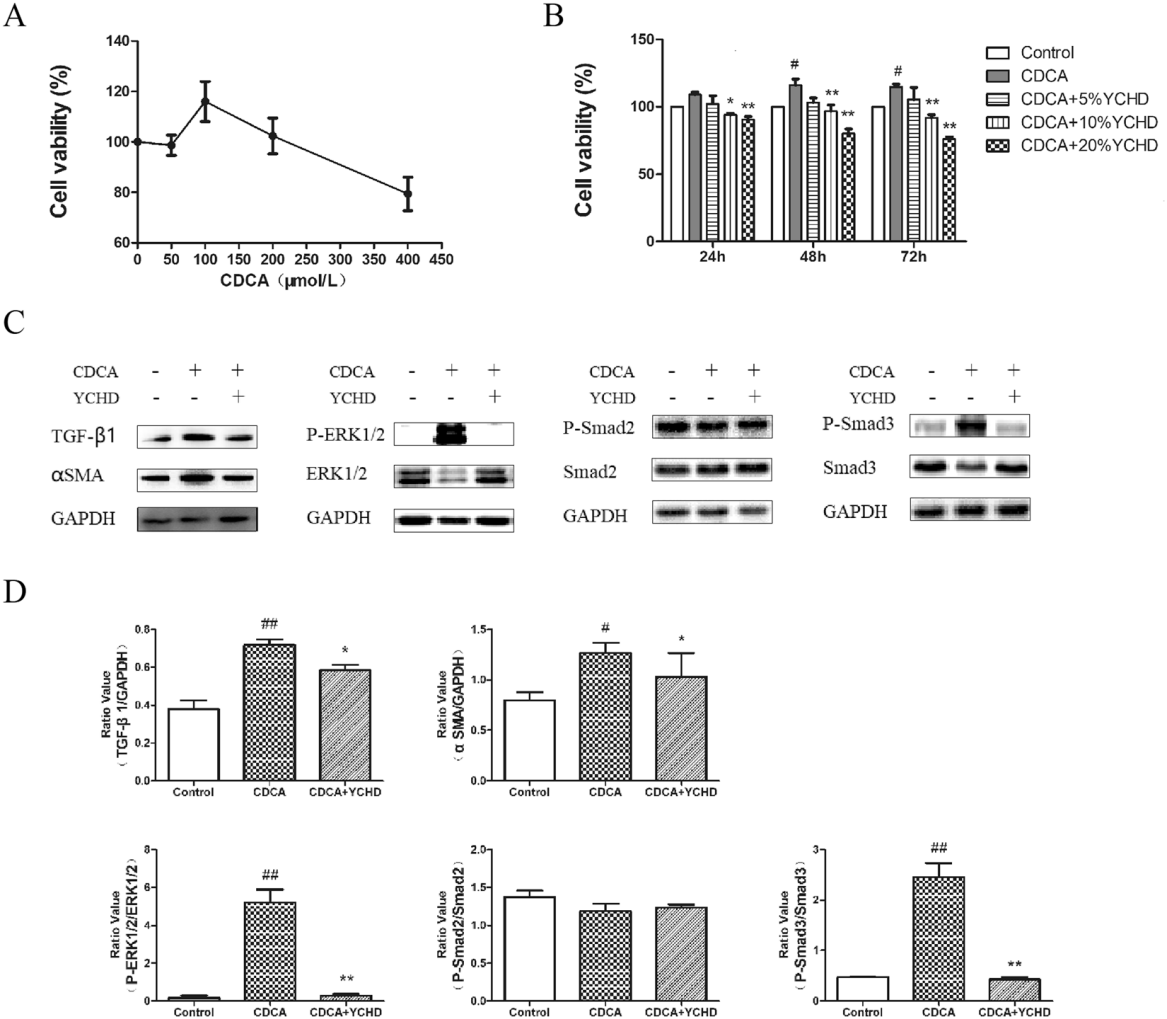
HSCs proliferation and activation exposed to CDCA and YCHD. (**A**) Viability of HSCs exposed to CDCA. (**B**)Viability of HSCs exposed to CDCA and YCHD. (**C**) Western blotting image for the expression of TGF-β1, α-SMA, p-Smad2, p-Smad3 and p-ERK1/2. (**D**) The histograms displayed relative abundance, represented by the band intensity in western blotting.

Excessive CDCA may represent an endogenous danger signal to initiate liver inflammation^8^; therefore, we used CDCA to induce proliferation in HSCs. As shown in Fig. 6A, cell viability was determined after exposing HSCs to CDCA at different concentrations. Because CDCA significantly increased the viability of HSC-T6 cells, subsequent experiments were performed with 100 μM CDCA. YCHD exposure significantly decreased the viability of HSC-T6 cells in a dose-dependent manner (Fig. 6B). CDCA significantly increased α-SMA, TGFβ1, p-Smad3, and p-ERK1/2 expression; however, they were decreased by YCHD treatment (Fig. 6C,D). Consequently, YCHD may inhibit cell proliferation and HSC activation by negatively regulating TGF-β1/Smad/ERK signalling pathways.

**Figure 7:**
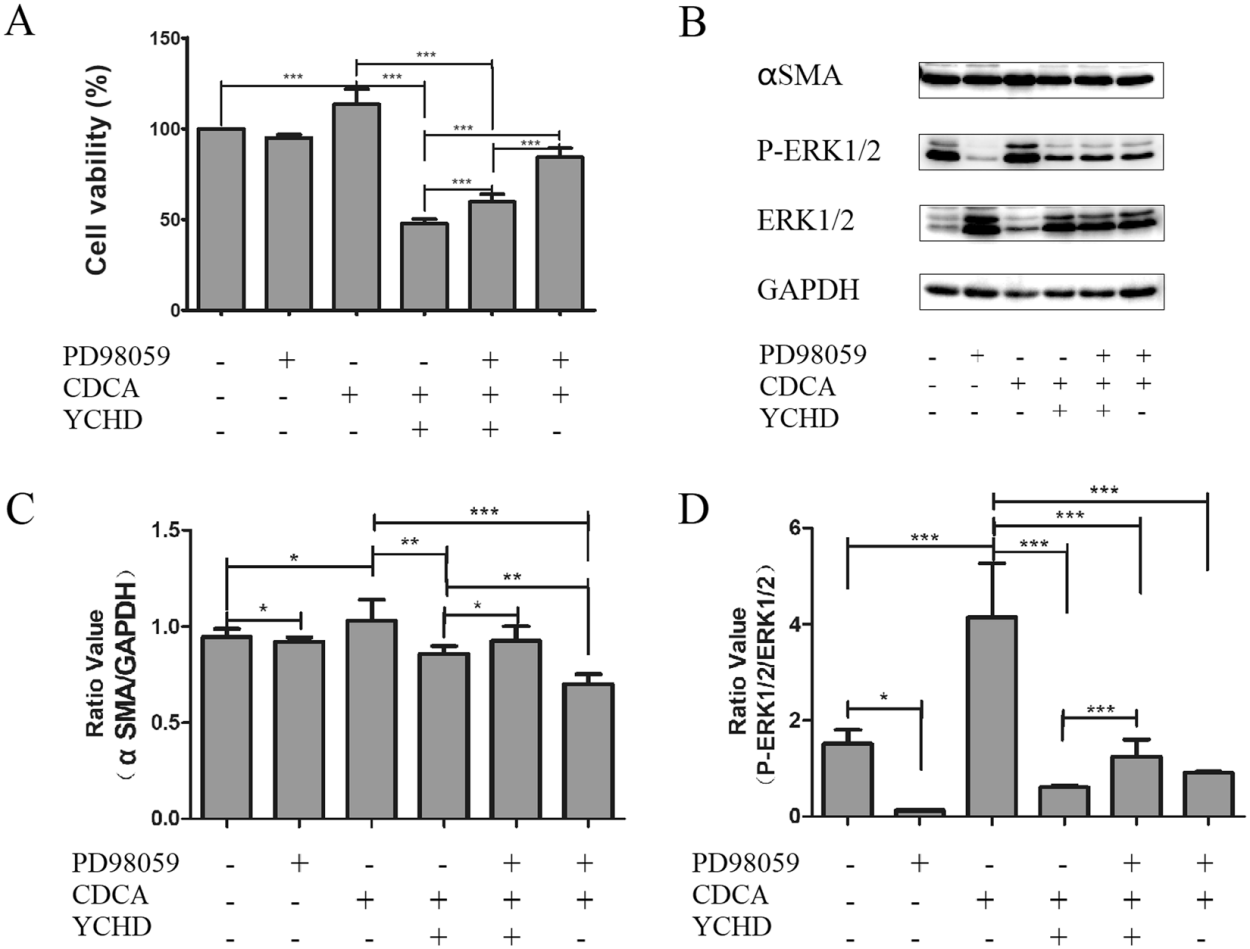
Effect of YCHD on the phosphorylation of ERK signaling pathway. (**A**) Viability of HSCs with or without PD98059, CDCA and YCHD. (**B**) Western blotting image for the expression of α-SMA and p-ERK1/2 in HSCs with or without PD98059, CDCA and YCHD. (**C** and **D**) The histograms displayed relative abundance, represented by the band intensity in western blotting.

To further clarify whether YCHD inhibits HSC proliferation and activation via TGF-β1/Smad/ERK signalling pathways, we measured the viability of HSCs and YCHD-induced expression of p-ERK1/2 protein in the presence of PD98059, an ERK inhibitor. Treatment with PD98059 and YCHD significantly enhanced the viability of CDCA-induced HSC-T6 cells compared with YCHD treatment (Fig. 7A). Additionally, the expression of α-SMA and p-ERK1/2 was significantly higher after YCHD and PD98059 treatment than after YCHD treatment alone (Fig. 7B–D). These findings indicate that YCHD could inhibit p-ERK1/2 to inhibit the proliferation and activation of HSCs.

## Discussion

Fibrosis is a common and serious complication of various diseases and occurs frequently. It is characterised by abnormal or excessive accumulation of ECM components, particularly fibrillar collagens, leading to disrupted tissue function in the affected organs^3,20–24^. Fibrosis is a key driver of progressive organ dysfunction in many inflammatory and metabolic diseases, including liver disease, pulmonary fibrosis, kidney disease, and heart failure^25–30^. TCM has been used since ancient times and was widely considered as an alternative or complementary therapy for the treatment of various diseases^31–35^. Although increasing evidence has shown that liver fibrosis can regress in many cases in the past decades^36^, there is a lack of clinically effective drugs for the treatment of cirrhosis. YCHD is a clinically effective treatment for liver and gallbladder dampness-heat syndrome in cholestatic liver diseases^18^. The intraperitoneal administration of DMN for 4 weeks could induce liver fibrosis and cause specific histological features of fibrotic liver tissue in animal model^37^. In the present study, we demonstrated that YCHD protects against pathological changes of liver fibrosis, and regulates hepatic Hyp content and the serum levels of ALT, AST, ALP, TBA, and TBIL. These data indicated that YCHD effectively reduced liver injury and fibrosis induced by DMN. Furthermore, we investigated the mechanisms of action of YCHD against liver fibrosis by analysing the metabolic profiles of bile acids.

Smit *et al*. hypothesised that toxic bile may cause hepatic injury^38^, and since then, the relationship between toxic bile acids and chronic liver injuries has been studied extensively. It has been reported that serum GCA, TCA, and TCDCA levels are higher in patients with obstructive cholestasis than in healthy individuals^39^. Patients with advanced cirrhosis also have increased levels of GCDCA, a toxic bile acid, compared with normal individuals^40^. The serum levels of CA, GCA, and TCA are significantly increased in animals with histopathological signs of hepatocellular necrosis, which were in accordance with the levels of standard biomarkers of liver injury^41^. In our study, the levels of free bile acids such as CA, DCA, CDCA, and HDCA; conjugated bile acids, especially TCDCA; and four glycine-conjugated bile acids were higher in the serum/hepatic tissues of DMN-induced liver fibrosis rats than in normal rats. This indicates that DMN increases the release of these bile acids from the liver into the blood. However, their levels were decreased in rats treated with YCHD compared with DMN-treated rats. This finding is consistent with the YCHD-induced improvement in liver function as shown by the levels of biochemical markers of liver injury, such as ALT, AST, ALP, TBA, and TBIL, indicating that YCHD decreases the release of toxic bile acids, including CA, DCA, CDCA, and GCDCA, into the blood stream in response to liver injury.

Bile acids are synthesised via two pathways. The initial step in the classical pathway of hepatic synthesis of bile acids is the enzymatic addition of a 7α hydroxyl group by CYP7A1 to form 7α-hydroxycholesterol, which is further hydroxylated by CYP8B1^42^. Alternatively, bile acid synthesis is initiated by CYP27A1. It has been reported that FXR activation in rats with established cirrhosis leads to the accelerated resolution of liver fibrosis^43^, and FXR expression in the rat liver significantly decreases in α-naphthylisothiocyanate (ANIT)-induced cholestasis model^44^. FXR induces the expression of SHP, which then binds to HNF4α and LRH-1 to inhibit the transcription of CYP7A1 gene. In this study, FXR, SHP, CYP7A1, CYP8B1, and CYP27A1 levels were upregulated in YCHD-treated rats, whereas they were downregulated in DMN-treated rats. This indicates that YCHD inhibits the synthesis of bile acids by regulating the negative feedback pathway.

The re-uptake of conjugated bile acids into hepatocytes occur through NTCP (SLC10A1) or OATP transporters^45^. NTCP (SLC10A1) mutation significantly elevates plasma levels of conjugated bile acids; however, it does not translate into liver injury, indicating that enterohepatic circulation of bile acids may be caused by other transporters, such as OATP, when NTCP is absent^46^. Bile acid excretion occurs across canaliculus and basolateral membranes into the duodenum or back into the systemic circulation via BSEP and MRP3^47,48^. In our study, MRP3 was activated in DMN-induced liver fibrosis rats, and the expression of NTCP, OATP2/3/4, and BSEP was significantly upregulated in YCHD-treated rats compared with that in DMN-treated rats. YCHD may increase bile acid reabsorption and restore the balance in bile acid excretion, thereby reducing the impact of toxic bile acids on liver injury and fibrosis in rats.

CDCA, which is considered to be potentially toxic, has been reported to induce mitochondrial injury and death of bile duct epithelial cells^49^ and to induce HSC proliferation and activation^19^. Hence, we explored the effect of YCHD on CDCA-induced HSC activation in this study. α-SMA is commonly used as an important marker of myofibroblast formation^50,51^, which reflects the activation of HSCs^52^. Therefore, we investigated α-SMA expression in HSC-T6 cells further. The results showed that CDCA could significantly upregulate α-SMA expression; however, the upregulated expression of α-SMA was decreased by YCHD treatment, indicating that toxic bile acids, such as CDCA, can induce HSC activation.

An interaction between the TGF-β1/Smad pathway and the ERK pathway has been established^53,54^, and TGF-β1/Smad/ERK signalling contributes to the progression of liver fibrosis^55^. In this study, we found that YCHD suppresses the protein expression of TGF-β1, p-Smad3 and p-ERK1/2, whereas DMN activates TGF-β1/Smad/ERK signalling to induce liver fibrosis in rats. To confirm these effects, we further quantified the expression of TGF-β1, p-Smad2, and p-ERK1/2 signalling in CDCA-activated HSCs. The results indicated that YCHD suppresses CDCA-induced HSC proliferation and activation by inhibiting TGF-β1/Smad/ERK signalling pathway.

Overall, this study demonstrated that YCHD prevents histopathological changes of hepatic dysfunction in DMN-induced liver fibrosis rats by regulating bile acid metabolism enzymes, which are associated with the increase in bile acid synthesis, reabsorption, and excretion. Furthermore, YCHD inhibits CDCA-induced HSC proliferation and activation by regulating TGF-β1/Smad/ERK signalling.

## Material and Methods

### Reagents

DMN and CDCA were purchased from Sigma-Aldrich (Saint Louis, MO, USA). CellTiter 96® AQueous One Solution Cell Proliferation Assay [3-(4,5-dimethylthiazol-2-yl)-5-(3-carboxymethoxyphenyl)-2-(4-sulfophenyl)-2H-tetrazolium; inner salt, MTS] was purchased from Promega (Madison, WI, USA). PD98059 and antibodies, including Smad2, p-Smad2, Smad3, p-Smad3, ERK1/2, and p-ERK1/2 were purchased from Cell Signaling Technology Inc. (Danvers, MA, USA); α-SMA and TGF-β1 were purchased from Abcam (Cambridge, MA, USA), and GAPDH was purchased from KangChen Biotech Inc. (Shanghai, China).

### YCHD Preparation

Granules of herbal concentrates comprising Yinchenhao (90 g), Zhizi (54 g), and Dahuang (36 g) were purchased from Shuguang Hospital. The granules were immersed in 1.2 L of cold water (6 mL/g of crude drug granules) for 30 min. This mixture was then boiled, slowly fried for 40 min, and filtered when hot under gravity. The remaining pellets were re-immersed in 0.8 L of water (4 mL/g of crude drug granules), boiled, slowly fried for 40 min, and filtered when hot under gravity. The filtrates were combined and concentrated to 400 mL at a final concentration of 630 mg/mL in a water bath at 95 °C. The fingerprint of YCHD was determined for quality control by HPLC^56^.

### Preparation of YCHD-medicated Serum

Sprague–Dawley rats were randomly divided into YCHD (n = 8) and vehicle control (n = 8) groups. Rats in the YCHD group received YCHD (3.15 g/kg, p.o.) twice a day for three days, whereas the control group received physiological saline (p.o.) twice a day for three days. One hour after the last administration, the rats were intraperitoneally anesthetised with amobarbital, and blood was sampled from the abdominal aorta and centrifuged. Aliquots of the separated serum were collected into 10-mL ampoules, and preserved at −80 °C until analysis.

This study was carried out in accordance with the recommendations of the Care and Use of Laboratory Animals published by the U.S. National Institutes of Health (NIH Publication No. 85-23, revised 1996) and the Animal Care and Use Committee of the Shanghai University of Traditional Chinese Medicine. The protocol was approved by the Animal Care and Use Committee of the Shanghai University of Traditional Chinese Medicine.

### Cell Culture

HSC-T6, a rat HSC cell line, was obtained from American Type Culture Collection (Manassas, VA, USA) and maintained in Dulbecco’s modified Eagle’s medium (Gibco, Life technologies, NY, USA) supplemented with 10% foetal bovine serum (Gibco, Life technologies, NY, USA), 100 U/mL penicillin, and 0.1 mg/mL streptomycin (Hyclone, Thermo Scientific, USA) at 37 °C in 5% CO_2_.

### Animal Experiments

Twenty-four 7-week-old male Wistar rats (200–250 g) were housed under standard conditions of temperature (17–25 °C) and light (12-h photoperiod). The rats were randomised into control (n = 9) and DMN-treated (n = 15) groups. In the DMN-treated group, 10 mg/kg DMN was administered intraperitoneally for three consecutive days each week for 4 weeks, and the rats in the control group received an equal volume of physiological saline. At the end of the second week, six DMN-treated rats were co-treated daily with YCHD (3.15 g/kg, p.o.), and the remaining nine rats received an equal volume of water. At the end of the fourth week, all animals were killed, and the liver and serum samples were collected for subsequent analysis. The study protocol was approved by the Animal Ethics Committee of Shanghai University of Traditional Chinese Medicine.

### Histological Analysis

Formalin-fixed liver tissues were processed, and 4-μm thick slices were stained with H&E and Sirius red. The specimens were observed for histopathological changes by light microscopy. Fibrosis was graded by three blinded pathologists according to the description by Scheuer^57^. Fibrosis scores were obtained after thorough examinations of three different areas of the tissue slide from each rat.

### Hepatic Hydroxyproline Content and Serum Biochemistry Analysis

Liver specimens (100 mg) were prepared for Hyp determination according to a modified method described by Jamall. The Hyp content served as an indirect measure of tissue collagen content and was weighed wet (μg/g). The serum levels of ALT, AST, ALP, Alb, and TBIL were measured according to the instructions provided by the manufacturers of corresponding analytical kits. TBA was measured at a clinical laboratory of Shuguang Hospital.

### Metabolic Profiling Analysis

Chromatographic analyses were performed with a Waters ACQUITY UPLC system using a ZQ2000 mass spectrometer. UPLC/MS-based metabolic profiling analysis of bile acids was carried out on serum samples according to established methods^58^.

### RT-qPCR

The expression of genes associated with bile acid metabolism was determined by RT-qPCR. Total RNA was extracted from liver tissues using the RNAsimple Total RNA Kit (TIANGEN, Beijing, China) and reverse-transcribed using the ReverTra Ace® RT-qPCR kit (TOYOBO, OSAKA, Japan). qPCR was performed on an ABI 7500 PCR System (Applied Biosystems, Foster City, CA, USA) under the following conditions: 95 °C for 30 s, 95 °C for 5 s (40 cycles); 60 °C for 30 s, and 72 °C for 15 s. The primer pairs used in this study are shown in Table S1. Each sample was run three times. The relative expression level of genes was calculated using beta-actin as the internal control.

### MTS Assay

HSCs were seeded in a 96-well plate at a density of 2.0 × 10^3^/well in triplicate to monitor cell viability. The cells were cultured for 1, 2, and 3 days at 37 °C. CellTiter 96 AQueous One solution (20 μL) containing MTS was added to each well. After 4 h of incubation at 37 °C, the absorbance at 490 nm was measured. Cell viability was calculated with respect to control samples.

### Western Blot Analysis

HSC cell lysates were collected, and the protein concentrations were determined by the BCA protein assay. Equal amounts of proteins were separated by sodium dodecyl sulphate polyacrylamide gel electrophoresis (SDS-PAGE) and transferred to polyvinylidene difluoride (PVDF) membranes as previously described^59^. Then, the membranes were incubated overnight at 4 °C with the primary antibodies, and GAPDH as an internal control. Subsequently, the membranes were incubated with a secondary antibody (Li-Cor Biosciences) for 1 h at room temperature. Finally, the target protein bands were visualised using the Li-Cor Odyssey scanner and software (Li-Cor Biosciences).

### Data Analysis

Statistical analysis was performed using the SPSS software (Chicago, IL, USA) and the GraphPad Prism software (San Diego, CA, USA). All values are expressed as the mean ± standard deviation (SD) and statistically analysed using one-way ANOVA. *P* < 0.05 was considered statistically significant.

## Electronic supplementary material

Supplementary Table and Figure

Supp Info
