# Supplementary material for: Yinchenhao Decoction Alleviates Liver Fibrosis by Regulating Bile Acid Metabolism and TGF-β/Smad/ERK Signalling Pathway
Source: Sci Rep. 2018 Oct 18;8:15367. doi: 10.1038/s41598-018-33669-4 (PMC6194075; doi:10.1038/s41598-018-33669-4)

Yinchenhao Decoction Alleviates Liver Fibrosis by Regulating Bile Acid Metabolism and TGF-β/Smad/ERK Signalling Pathway

Fei-Fei Cai[1], Rong Wu[1], Ya-Nan Song[1], Ai-Zhen Xiong[2], Xiao-Le Chen[1], Meng-Die Yang[1], Li Yang[2], Yuanjia Hu[3], Ming-Yu Sun[4]*, Shi-Bing Su[1]*

[1]Research Center for Traditional Chinese Medicine Complexity System, Shanghai University of Traditional Chinese Medicine, Shanghai, China

[2]The MOE Key Laboratory for Standardization of Chinese Medicines, Institute of Chinese Materia Medica, Shanghai University of Traditional Chinese Medicine, Shanghai, China

[3]State Key Laboratory of Quality Research in Chinese Medicine Institute of Chinese Medical Sciences, University of Macau, Macao SAR, China

[4]Liver disease institute, Shuguang Hospital, Shanghai University of Traditional Chinese Medicine, Shanghai, China

Supplementary Table S1:

Table S1 Primer pairs used for qRT-PCR.

Gene		Primer Sequence (5'-3')
FXR	F	TCCTCGTCCTATTATTCCAACC
	R	CTCATCCCCTTTTATTCTTCCC
SHP	F	CTCGGTTTGCATACAGTGTTTGAC
	R	GCATATTGGCCTGGAGGTTTT
LRH-1	F	GCTGCCCTGCTGGACTACAC
	R	TGTAGGGCACATCCCCATTC
HNF-4α	F	CCAGCCTACACCACCCTGGAGTT
	R	TTCCTCACGCTCCTCCTGAA
CYP7A1	F	ACATGGAGAAGGCCAAGACG
	R	GGCAGGTCATTCAGTTGCAC
CYP8B1	F	AGTACACATGGACCCCGACATC
	R	GGGTGCCATCAGGGTTGAG
CYP27A1	F	GGAAGGTGCCCCAGAACAA
	R	GCGCAGGGTCTCCTTAATCA
OATP2	F	GGAAAATCCACTGCTGAGGTAG
	R	TCAGACAGGCAGAGCCAGAATG
OATP3	F	CAAGGAGGAGAAACACAGAGAA
	R	TTGGGCAAGAAGGTAAACATAT

OATP4	F	CCAAAACAGATGAGGAGAAGAA
	R	TGAACAGGTAAGTAAAGGAGCC
NTCP	F	CTCTCTTCCAACTCAATCCAAG
	R	TGATGATGAGAAGTCCTTCTGC
MRP3	F	ATACCAACACTCCAGACCTCAC
	R	AGATACACCACAGCAGAACACC
BESP	F	CTGGCTGATGCTTATGGGAGGC
	R	TCTGGTGGAAGGAGCTGTTGAT
ACTB	F	ACGTTGACATCCGTA
	R	CTGGAAGGTGGACAGTGAG

Supplementary Figure S1:

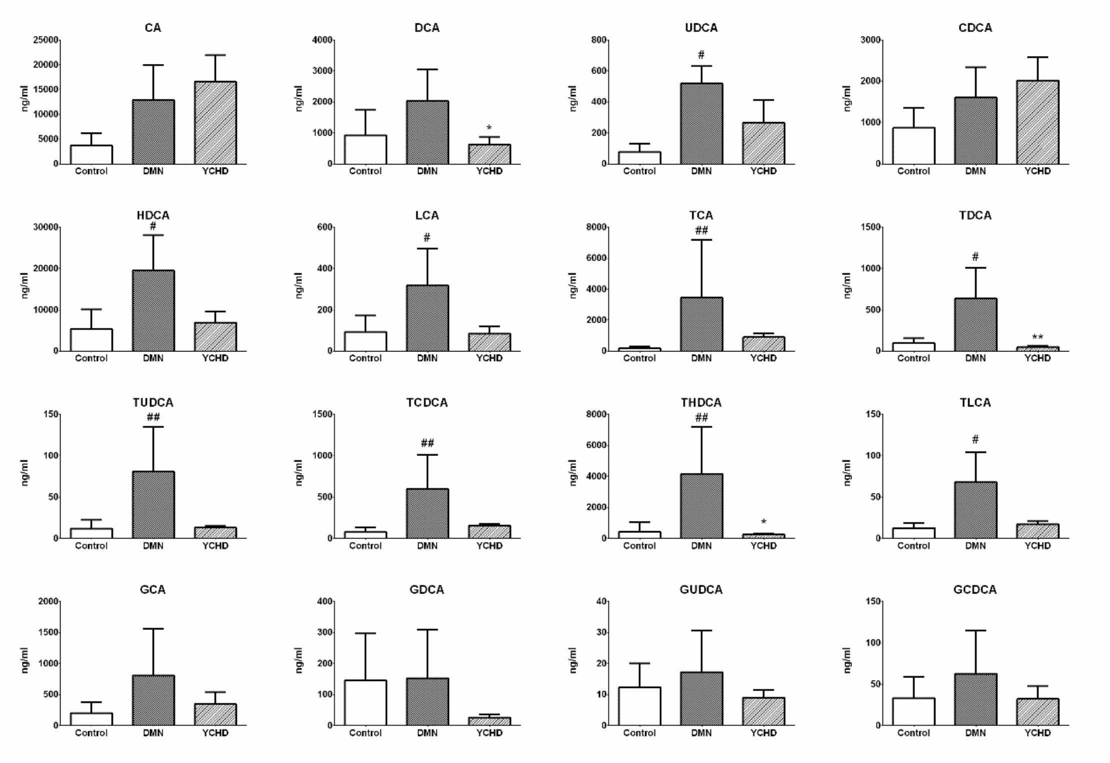

Fig. S1. Metabolic profiling of bile acids in serum after treated by YCHD.

Supplementary Figure S2:

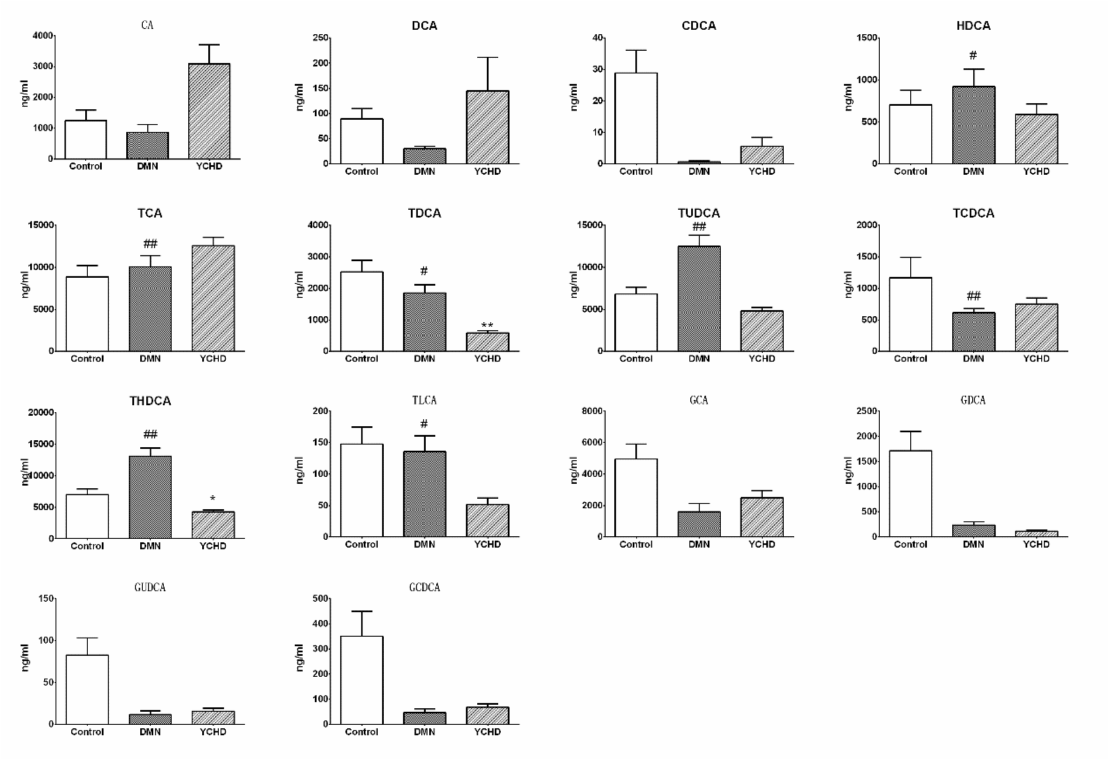

Fig. S2. Metabolic profiling of bile acids in hepatic tissues after treated by YCHD.

Supplementary Figure S3:

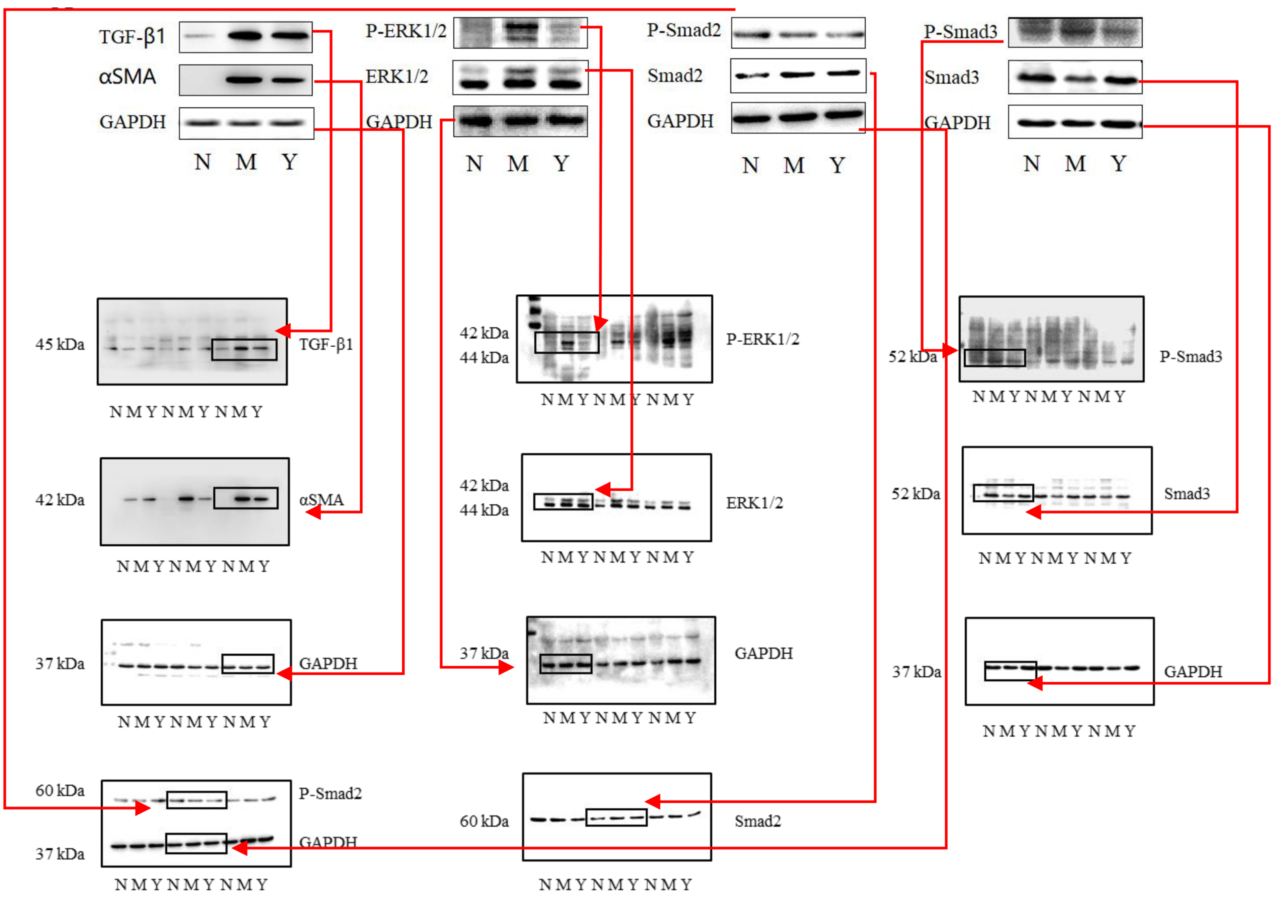

Supplementary Figure S4:

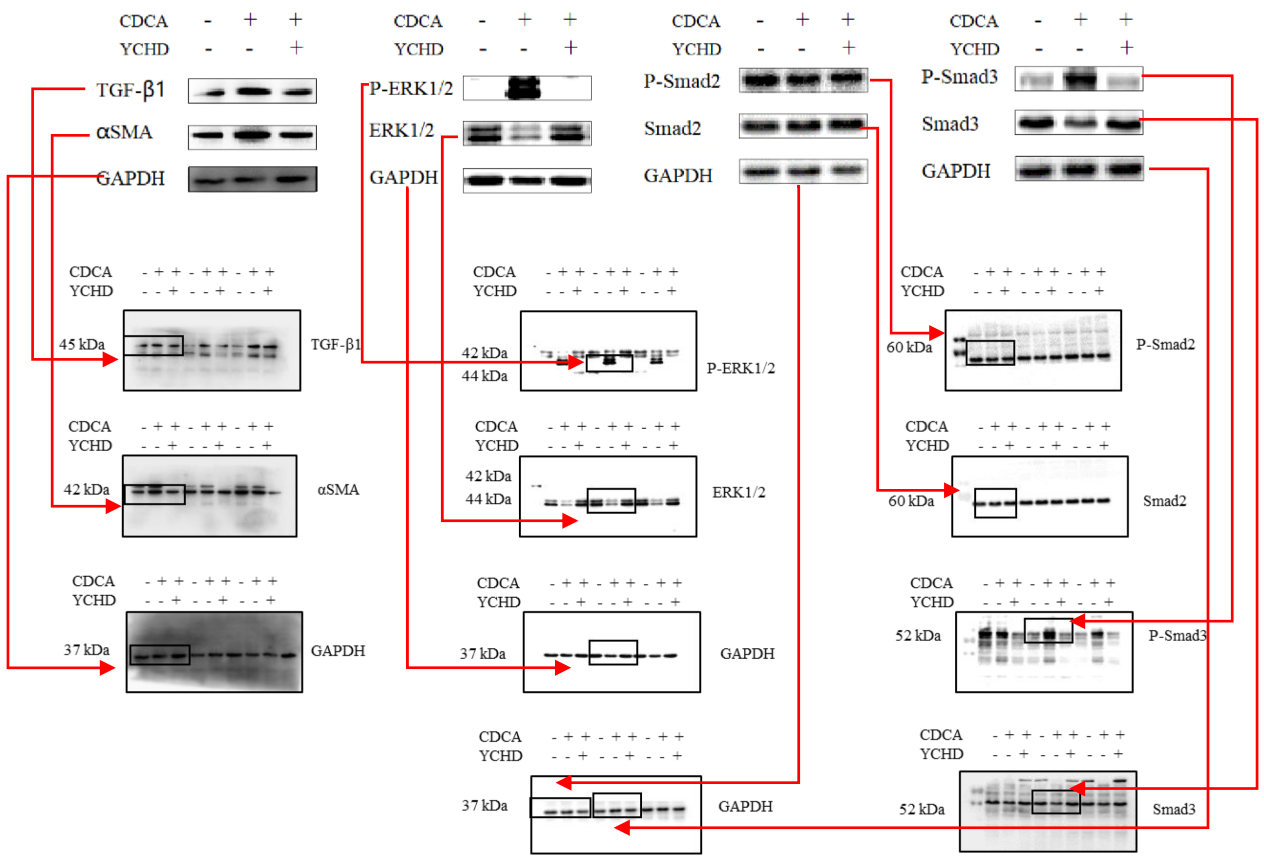

Supplementary Figure S5:

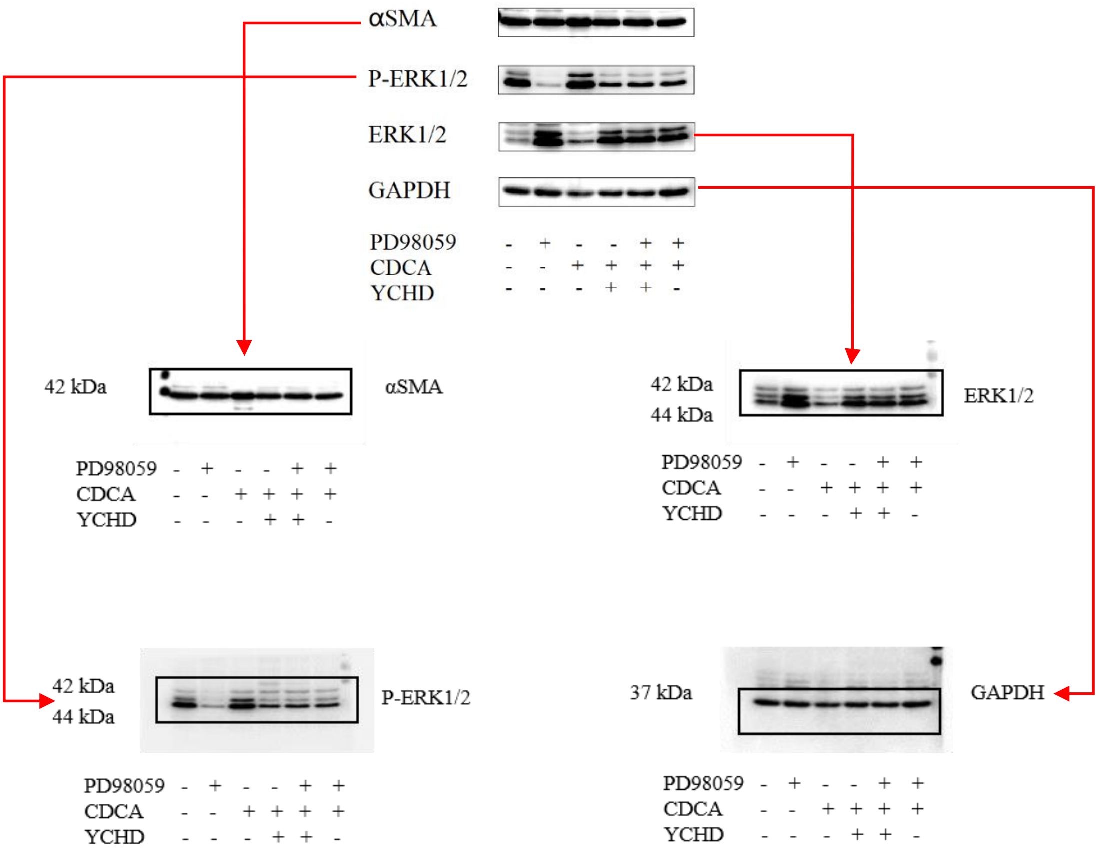

Supplement: Supplementary file 1 — Supplementary Table and Figure [file 41598_2018_33669_MOESM1_ESM.pdf]
