# Supplementary material for: Yinchenhao Decoction Alleviates Liver Fibrosis by Regulating Bile Acid Metabolism and TGF-β/Smad/ERK Signalling Pathway
Source: Sci Rep. 2018 Oct 18;8:15367. doi: 10.1038/s41598-018-33669-4 (PMC6194075; doi:10.1038/s41598-018-33669-4)
Supplement: Supplementary file 2 — Supp Info [file 41598_2018_33669_MOESM2_ESM.pdf]

Analysis of fibrosis in control or pressure overloaded rat hearts after mechanical unloading by heterotopic heart transplantation

Andreas Schaefer[1,2,8,*], MD, MHBA; Yvonne Schneeberger[1,2,8,*], MD, MHBA; Steven Schulz[2,3,8]; Susanne Krasemann[4], PhD; Tessa Werner[2,3,8], PhD; Angelika Piasecki[3]; Grit Höppner[3]; Christian Müller[2,5], Kristina Lorenz[6], PhD; David Wieczorek[7], PhD; Alexander P Schwoerer[2,8,9], MD, MME; Thomas Eschenhagen[2,3,9], MD; Heimo Ehmke[2,8,9], MD; Hermann Reichenspurner[1,2,9], MD, PhD; Justus Stenzig[2,3,9#], MD, PhD; Friederike Cuello[2,3,9#], PhD

[1]Department of Cardiovascular Surgery, University Heart Center Hamburg; [2]DZHK (German Centre for Cardiovascular Research) partner site Hamburg/Kiel/Lübeck; [3]Department of Experimental Pharmacology and Toxicology, University Medical Center Hamburg-Eppendorf; [4]Institute of Neuropathology, University Medical Center Hamburg-Eppendorf; [5]Department of General and Interventional Cardiology, University Heart Center, Hamburg, Germany; [6]Comprehensive Heart Failure Center, Würzburg; Leibniz-Institut für Analytische Wissenschaften-ISAS-e.V. Dortmund; West German Heart and Vascular Center, Essen; [7]Cardiovascular Center, University of Cincinnati, Ohio, USA; [8]Department of Cellular and Integrative Physiology, University Medical Center Hamburg-Eppendorf; [9]Cardiovascular Research Center, University Medical Center Hamburg-Eppendorf

*AS and YS contributed equally to the work; [#]JS and FC contributed equally to the work

Total word count: 8056

Corresponding author:
Andreas Schaefer, MD, MHBA
University Heart Center Hamburg, Martinistrasse 52, 20246 Hamburg, Germany
Tel.: + 49 40 7410 52440
Fax.: +49 040 7410 54931
Email: and.schaefer@uke.de

Abstract

Background: Mechanical unloading (MU) by implantation of left ventricular assist devices (LVAD) has become clinical routine. This procedure has been shown to reverse cardiac pathological remodeling, with the underlying molecular mechanisms incompletely understood. Most studies thus far were performed in non-standardized human specimens or MU of healthy animal hearts. Our study investigates cardiac remodeling processes in sham-operated healthy rat hearts and in hearts subjected to standardized pathological pressure overload by transverse aortic constriction (TAC) prior to MU by heterotopic heart transplantation (hHTx/MU).

Methods: Rats underwent sham or TAC surgery. Disease progression was monitored by echocardiography prior to MU by hHTx/MU. Hearts after TAC or TAC combined with hHTx/MU were removed and analyzed by histology, western immunoblot and gene expression analysis.

Results: TAC surgery resulted in cardiac hypertrophy and impaired cardiac function. TAC hearts revealed significantly increased cardiac myocyte diameter and mild fibrosis. Expression of hypertrophy associated genes after TAC was higher compared to hearts after hHTx/MU. While cardiac myocyte cell diameter regressed to the level of sham-operated controls in all hearts subjected to hHTx/MU, fibrotic remodeling was significantly exacerbated. Transcription of pro-fibrotic and apoptosis-related genes was markedly augmented in all hearts after hHTx/MU. Sarcomeric proteins involved in excitation-contraction coupling displayed significantly lower phosphorylation levels after TAC and significantly reduced total protein levels after hHTx/MU.

Conclusions: Development of myocardial fibrosis, cardiac myocyte atrophy and loss of sarcomeric proteins was observed in all hearts that underwent hHTX/MU regardless of the disease state. These results may help to explain the clinical experience with low rates of LVAD removal due to lack of myocardial recovery.

Abbreviations

AA	Ascending aorta
BW	Body weight
CF	Continuous flow
H&E	Hematoxylin & eosin staining
hHTx	Heterotopic heart transplantation
LV	Left ventricle
LVAD	Left ventricular assist device
MU	Mechanical unloading
PA	Pulmonary artery
PSR	Picrosirius Red staining
TAC	Transverse aortic constriction
TTE	Transthoracic echocardiography
WB/IB	Western immunoblot analysis

Introduction

Due to limited availability of donor organs for heart transplantation, implantation of intracorporal miniaturized left ventricular assist devices (LVAD) became clinical daily routine, with the number of procedures increasing annually (1, 2). Next-generation LVAD systems virtually all follow a continuous-flow (CF) principle by constantly moving blood from the left ventricle (LV) to the aorta and thereby mechanically unloading the heart (3). Cardiac mechanical unloading (MU) of the LV by reducing myocardial volume and consequently pressure overload is considered to reverse remodeling of the myocardium (4). However, reports are scarce that describe successful myocardial recovery under LVAD therapy permitting subsequent LVAD explantation (5, 6). Molecular analyses of explanted hearts after MU reveal multifaceted processes of reverse remodeling. A decline of cardiac hypertrophy, recovery of β-adrenergic receptor expression, improved calcium cycling and alterations in the gene expression profile were observed (7, 8). Additionally, the contribution of an altered inflammatory response and the generation of reactive oxygen species have been described (9). Nevertheless, thus far neither can a definitive beneficial clinical outcome in terms of myocardial recovery under MU be predicted nor have the drivers of myocardial recovery been identified (10, 11). One factor that may contribute to this controversy on the grounds of inconclusive results is that molecular investigations of myocardium after MU are often performed in human heart tissue derived from explanted hearts (12, 13). Heart tissue of LVAD patients can be poorly standardized due to different origins of heart failure and varying LVAD therapy duration. To strategically investigate the molecular mechanisms underlying reverse remodeling after MU, an animal model is indispensable, such as the heterotopic heart transplantation in rat previously described by Ono and Lindsey (14). Here, MU was induced by connecting the ascending aorta (AA) and pulmonary artery (PA) of the donor organ to the abdominal aorta and the inferior vena cava of the recipient animal. In this vascular configuration, blood flow into the left and right ventricle is largely absent, reducing pressure and volume load of the ventricles. Coronary perfusion, however, is preserved,

leading to a transplanted “in vivo Langendorff” heart. Effects of MU can be adequately assessed in this animal model.

Thus far, MU has often been performed with healthy rat hearts, not representing the typical heart failure pathology (15-17). To better reflect the clinical situation, however, previous studies have also assessed the outcome of MU in volume-overloaded hearts (18) or in a genetic model of dilated cardiomyopathy (19). To further complement these studies with a clinically relevant model, we set out to investigate the molecular mechanisms of MU-mediated pathological remodeling in pressure-overloaded hearts. To this end, we subjected rat hearts to 3 or 6 weeks of thoracic aortic constriction (TAC) prior to heterotopic heart transplantation and investigated alterations in intracellular signaling mechanisms. We thus aimed to use our standardized protocol for pressure overload/MU to establish a possible relationship between pre-MU disease-severity and outcome after MU.

Material and methods

Materials

Anti-cardiac myosin-binding protein C (cMyBP-C) antibody and anti-CTGF were purchased from Santa Cruz Biotechnology (Dallas, TX, USA), cMyBP-C-pSer282 antibody from Enzo Life Sciences (Lörrach, Germany (15)), anti-phospholamban (PLN) and PLN-pSer16 antibody from Badrilla (Leeds, UK), anti-p44/42 MAPK (ERK) and pThr202/Tyr204-p44/42 MAPK (ERK) from Cell Signaling Technology (Frankfurt, Germany) and anti-tropomyosin (TM1) sarcomeric antibody was from Sigma-Aldrich (Taufkirchen, Germany). pSer283-TM1 antibody was a kind gift from Dr. David Wieczorek (Cardiovascular Research Center, Cincinnati, USA).

Study design

To investigate potential differences in MU-mediated remodeling between sham-operated healthy control hearts and hearts undergoing TAC-induced pressure overload prior to heterotopic heart transplantation, a novel rat model combining TAC and heterotopic heart transplantation (hHTx) was employed (16). Following sham (**group 1**) or TAC surgery for the duration of 3 (**group 2**) or 6 weeks (**group 3**), disease progression in groups 2 and 3 was monitored by echocardiography (in vivo) as compared to sham-operated hearts. Three or four hearts per group (sham-operated or TAC-operated hearts, for 3 weeks or 6 weeks) were then heterotopically transplanted and mechanically unloaded for 14 days (**group 4, 5, 6**). At the indicated points in time, animals were sacrificed and the hearts removed and analyzed by histology (group 1 n=10; group 2 n=10; group 3 n=8; group 4-6 n=3-4), western immunoblot analysis and with regard to gene expression (group 1-3 n=4-8; group 4-6 n=3).

The group design is illustrated schematically in **Figure 1**.

Animal experiments

All experiments were conducted in accordance to local institutional guidelines after approval by local authorities (Institutional Animal Care and Use Committee: Behörde für Gesundheit

und Verbraucherschutz, Hamburg, Germany; File reference: G82/14). All animals included in the study received humane care in compliance with the "Principles of Laboratory Animal Care" formulated by the National Society for Medical Research and the "Guide for the Care and Use of Laboratory Animals" prepared by the Institute of Laboratory Animal Resources. Biometrical planning using G-Power software (v. 3.1.7, Kiel University) was used to reduce animal numbers. Buprenorphine and carprofen were used to ameliorate suffering during surgery. Animals were sacrificed by an overdose of pentobarbital and subsequent decapitation.

Transverse aortic constriction (TAC)

This study was performed in animals undergoing a combination of TAC surgery with subsequent heterotopic heart transplantation (hHTx) as previously described (17). To this end, TAC surgery was performed in 3 week-old (40–50 g) male syngenic Lewis rats (Charles River Germany GmbH, Sulzfeld, Germany) to induce pressure overload. Animals were anaesthetized by intubation and ventilation with 2%-Isoflurane in oxygen (0.7 ml/100 g bodyweight (BW), 90 strokes/min). With the animals placed in a supine position, the underlying sternum was excavated after a 2 cm median cut along the neck. After median hemisternotomy, the sternum halves were kept apart with 4–0 vicryl sutures (Ethicon Inc., Somerville, NJ, USA). The aorta was dissected from the pulmonary trunk and a titanium clip was placed on the aortic arch between the brachiocephalic trunk and the left common carotid artery. The clip was delivered by a clip applicator (WECK, Horizon, Metal Ligation Sytem, Teleflex, Morrisville, NC, USA), with an adjustable screw, allowing for a remaining internal diameter of 0.45 mm after closure. The sternum and the skin were then closed and the animals were kept on a heating mat until they had reached full consciousness.

Echocardiography

Two weeks after the initial TAC surgery, all animals were examined echocardiographically (Vevo 770 system, 20 MHz center frequency single element transducer, VisualSonics Inc.,

Toronto, Canada). The systolic pressure gradient across the stenosis was evaluated by color duplex sonography and only animals with a maximum gradient above 50 mmHg were included in the study. Three weeks after the procedure animals showed myocardial hypertrophy with preserved fractional shortening (FS) and six weeks after TAC thinned myocardium with reduced FS.

Heterotopic heart transplantation (hHTx)

TAC-induced pressure overload was maintained for three weeks (**group 2**) to induce hypertrophy or 6 weeks (**group 3**) to mimic heart failure. HHTx was performed on 220–250 g Lewis rats. To reduce myocardial burden in the pre-impaired heart, donor and recipient animals were anesthesized with sevoflurane (SEVOrane, Abbott Laboratories Inc., Chicago, IL, USA). Analgesia was administered using 0.04 mg/kg BW buprenorphine (Temgesic, Reckitt Benckiser, Slough, Berkshire, UK) and 4–5 mg/kg BW carprofen s.c. After opening of the abdomen 500 I.U. heparin were injected into the inferior vena cava of the donor rat. The chest was opened in a butterfly fashion with median sternotomy and cutting at the height of the diaphragm arch. Subsequently, topical cooling with ice-cold saline and rapid administration of cardioplegia (St. Thomas-Hospital solution I, Dr. Franz Köhler Chemie GmbH, Bensheim, Germany) was performed. After loss of contractility and collapse of coronary arteries, vena cava superior and inferior as well as pulmonary veins were ligated using Mersiline (Ethicon Inc., Somerville, NJ, USA). Transplantation was performed as described by Ono and Lindsey (14), involving anastomosing of the ascending aorta to the recipient rat's abdominal aorta and the pulmonary artery to the abdominal part of the recipient rat's vena cava inferior using Prolene 8–0 (Ethicon Inc., Somerville, NJ, USA). Due to the syngenic nature of the utilized Lewis rats, no immunosuppression had to be performed. After 14 days of mechanical unloading, with daily digital testing of graft contractility, animals were euthanized and grafts were explanted for histological and molecular analyses.

Immunohistochemistry

Tissues were fixed in 4% buffered formalin and processed for paraffin embedding. Sections were cut (2 µm) and stained with hematoxylin and eosin according to standard procedures. For analysis of fibrosis, samples were stained with Picrosirius Red solution (Sirius Red dye dissolved in picric acid) according to standard procedures. For the detection of dystrophin, heat-mediated antigen retrieval was performed and samples were incubated with anti-dystrophin antibody (clone1808, 1:200, MAB1645, EMD Millipore). For detection of specific binding, the Ultra View Universal DAB Detection Kit (Ventana, Roche) was used, which contains secondary antibodies, DAB stain and counter staining reagent for detection of nuclei. Images were taken with a Leica DMD108 digital microscope. The quantification of fibrosis and cell diameter in tissue sections were carried out in a blinded fashion with ImageJ software (20).

Preparation of rat heart tissue samples for western immunoblot

Rat heart tissue was snap-frozen in liquid N_2, powderized and homogenized as a 1% homogenate (w/v) in a buffer consisting of 100 mmol/l Tris pH 7.4 and protease inhibitors (Complete, Roche). 3x reducing Laemmli sample buffer supplemented with 9% (v/v) β-mercaptoethanol was used. The samples were boiled for 5 min at 95 °C.

Assessment of collagen content

To assess total collagen in heart samples from animals from groups 1, 2 and 3, a colorimetric assay was used (Soluble Collagen Assay, Cellbiolabs). To this end, hearts from 8 animals per group were transversally cut below the papillary muscles. The basal section was used for histological analysis while the apical part was powderized in liquid N_2. The powder was used in a 1/20 dilution for the colorimetric assay according to the manufacturer's instructions. Absorption was analyzed on a Safire^2 plate reader (Tecan).

Western immunoblot analysis

Western immunoblot analysis was carried out as described previously (21). In brief, tissue homogenate samples were separated by SDS-PAGE (7.5-15%) and transferred to polyvinylidene difluoride (PVDF) or nitrocellulose membrane. After blocking non-specific binding sites with 10% (w/v) non-fat skimmed milk or 5% bovine serum albumin in 0.1% (v/v) Tween 20-TBS (in mmol/l: Tris 20, NaCl 137; pH 7.6), membranes were incubated overnight at 4 °C with primary antibodies, followed by horseradish peroxidase (HRP)-conjugated secondary antibodies on the next day. Specific protein bands were detected by enhanced chemiluminescence (GE Healthcare) and quantified using the Gene Tools software (Syngene).

Gene expression analysis

Gene expression was analyzed by qPCR and NanoString technology from RNA prepared from whole LV tissue. QPCR was performed to monitor disease progression in response to pressure overload and NanoString analysis was employed to extensively investigate the molecular cause of the fibrotic remodeling processes. For both purposes, RNA was extracted from LV tissue (RNeasy mini kit, QIAGEN, Hilden, Germany). For subsequent qPCR analysis, RNA was reversely transcribed (High Capacity cDNA Reverse Transcription kit, Applied Biosystems, Foster City, CA, USA). CDNA concentration was quantified by qPCR (HOT FIREPol EvaGreen qPCR Mix Plus, Solis BioDyne, Tartu, Estonia) followed by calculation using the delta-delta-ct-method. Ct values were normalized to glucuronidase beta (Gusb) as a common housekeeping gene. Primers can be found in **Supplementary Table 1**. For gene expression analysis with the fluorescence-based NanoString (Redwood City, CA, USA) single RNA molecule counting technology, an in-house designed TagSet comprising 27 genes coding for proteins involved in excitation-contraction coupling and transcripts with known deregulation in pathological cardiac hypertrophy was adopted. 50 ng RNA per sample were hybridized to target specific capture and reporter probes at 67 °C overnight (16 h) according to the manufacturer's instructions. Samples were cooled down to 4 °C, loaded into

the NanoString cartridge and the nCounter Gene Expression Assay was started immediately. Raw data (**Supplementary Table 2**) was analyzed with the proprietary nSolver Data Analysis Software (NanoString) including background subtraction using negative controls and normalization to 6 different housekeeping genes (ATP binding cassette subfamily F member 1 (Abcf1), actin beta (Actb), clathrin heavy chain (Cltc), glyceraldehyde-3-phosphate dehydrogenase (Gapdh), phosphoglycerate kinase (Pgk1), tubulin beta 5 class 1 (Tubb5)).

Statistical analysis

Statistical comparisons were performed by One-way ANOVA followed by Dunnett's post-test for multiple comparisons unless otherwise stated. Echocardiographic data were analyzed by Student's t-test. Quantitative data are presented as mean±SEM in bar charts, as mean±SD in the summary table (**Table 1**) and $P<0.05$ was considered significant. To account for batch effects in cell diameter measurements (**Figure 4B**, 20 cells each from 3 animals per group), we used the clustered Generalized Estimation Equation (GEE) to test for statistical significance. The 'gee' software package for R-software version 3.4.1 was used for calculation (R Development Core Team; Carey et al. (2015) gee: Generalized Estimation Equation Solver. R package version 4.13-19).

Results

TAC surgery and hHTx

As depicted in **Figure 1**, 10 animals (30-40 g; 3 weeks old) underwent sham surgery and thus constitute the sham-control group (**group 1**). In total, 30 animals underwent clip-based TAC surgery. Of them, 10 animals were included into the 3 weeks TAC group (“hypertrophy”; **group 2**). In this group, all animals survived the 3 weeks time-interval of pressure overload until organs were harvested (10/10, 100%). For the 6 weeks TAC group (“heart failure”; **group 3**), another 10 animals were considered, of which 8/10 animals (80%) survived the 6 weeks time-interval. Two animals died of progressive heart failure. The remaining 10 animals were operated to constitute the TAC/MU groups. The hearts of these TAC operated animals were subjected to heterotopic heart transplantation 3 or 6 weeks after surgery, respectively. Of these, 7/10 (70%) survived the procedure. These 7 animals formed the mechanically unloaded diseased heart group (4 hearts after 3 weeks of TAC in **group 5**; 3 hearts after 6 weeks of TAC in **group 6**) and were compared to an additional 3 sham-operated mechanically unloaded hearts (**group** 4).

Echocardiography and clinical phenotyping

Detailed functional parameters and clinical results obtained by echocardiography are summarized in **Table 1**. All animals were examined by echocardiography 2 weeks after sham or TAC surgery. All TAC operated animals displayed the desired pressure gradient across the stenosis, of which animals after 3 weeks of TAC (**group 2**) presented higher pressure gradients compared to after 6 weeks of TAC (**group 3**; 72.7±22.7 vs. 50.9±4.1 mmHg Δp^{max}; *P*=0.02; 24.4±7.3 vs. 17.3±1.5 mmHg Δp^{mean}; *P*=0.02). In both groups, animals displayed significant hypertrophy and decreased systolic function after 3 and 6 weeks, respectively. Compared to sham-operated control rats, left ventricular inner surface in the transversal echo plane decreased to 5.8±1.0 mm² (vs. 9.6±2.4 mm²; *P*<0.001) after 3 weeks of pressure overload with an impaired fractional shortening (FS) of 23.7±4.3% vs. 28.2±1.5% (*P*<0.01). After 6 weeks of clip application, systolic function was lower with a remaining fractional

shortening of only 17.1±3.2% vs. 28.2±1.5% ($P<0.0001$) and left ventricular inner surface had increased to 19.9±1.1 mm² ($P<0.001$). Moreover, ejection fraction, as again determined by transthoracic echocardiography, differed significantly between groups 1 to 3 (52.1±2.3% vs. 33.8±4.5%; $P<0.0001$ vs. 20.5±16.1%; $P=0.02$) Furthermore, animals presented clinical signs of heart failure such as dyspnea, rough fur and pale-bluish limbs 6 weeks after intervention.

Histological analysis after TAC-induced pressure overload and subsequent hHTx/MU

Disease progression in response to pressure overload (**Figure 2**) and subsequent hHTx with mechanical unloading for 2 weeks (**Figure 3**) was monitored histologically by staining with hematoxylin and eosin (H&E) to visualize morphological changes, Picrosirius Red (PSR) staining and colorimetric collagen assay to investigate fibrosis, and dystrophin staining to analyze changes in cardiac myocyte diameter. Representative images of transversal sections of hearts subjected to pressure overload for 3 and 6 weeks both revealed wall thickening and heart dilation, accompanied by mild fibrotic remodeling when compared to sham-operated control hearts (**Figure 2A, B, C**). Interestingly, analysis of transversal sections of diseased hearts after hHTx/MU intervention exhibited a significantly reduced overall surface area in comparison to hearts subjected to TAC surgery without subsequent hHTx/MU intervention (**Figure 3, 4A**). As expected, cardiac myocyte diameter assessed by dystrophin staining was significantly higher in the TAC groups. HHTx/MU led to a regression of the cardiac myocyte diameter to levels observed in sham-operated control hearts (**Figure 4B**). Importantly, PSR-staining of heart sections from diseased hHTx/MU hearts demonstrated exacerbated fibrotic remodeling, which was also detectable in sham-operated hHTx/MU hearts (**Figure 4C**). This suggests that mechanical unloading of rat hearts for two weeks activates a fibrotic remodeling program regardless of the disease status of the donor heart.

Gene expression

To investigate whether distinct alterations in gene expression induced by pressure overload and mechanical unloading are causally related to the remodeling processes observed by histology, gene expression was analyzed by qPCR and NanoString technology. Due to the small number of samples within group 5 and 6, the gene expression data were combined and labeled group TAC/hHTx/MU. Initial qPCR analysis was performed for common hypertrophy associated genes (**Figure 5**). As expected, the hypertrophic gene program was activated in both TAC groups. Acta1 (alpha 1 skeletal muscle actin) and Myh7 (beta-myosin heavy chain) were significantly higher expressed than in the sham-operated control group. These results are in line with the previous functional, phenotypical and histological data.

In the hHTx/MU groups, most differences in gene expression did not reach statistical significance due to small group sizes and large heterogeneity between replicates, attributable to the complex surgical transplantation procedure. However, expression of Acta1, My6 (alpha-myosin heavy chain), Atp2a2 (sarcoplasmic/endoplasmic reticulum Ca^{2+} ATPase 2), Nppa (natriuretic peptide A) and Nppb (natriuretic peptide B) were found to be markedly lower in both the sham-operated and the TAC hHTx/MU groups than in sham-operated controls without hHTx/MU intervention. Also Col1a1 (collagen type I alpha 1 chain) expression remained high in the hHTx/MU groups, which could contribute to the observed fibrotic remodeling. Interestingly, no obvious difference in gene expression of commonly assessed pro-hypertrophic genes was observed between the sham-operated hHTx/MU group and the TAC/hHTx/MU group, which was consistent with the histological observations.

By NanoString technology, a panel of 27 transcripts was analyzed encoding structural cardiac myocyte proteins (Acta1, Acta2 (alpha 2 skeletal muscle actin), Actc1 (cardiac muscle alpha actin), Actn2 (alpha actinin 2), Atp2a2, Casq2 (calsequestrin 2), Myh6, Myh7, Nppa, Nppa, Pln (phospholamban), Ryr2 (ryanodine receptor 2)), transcription factors (Fhl1 (four-and-a-half LIM domains 1), Fhl2 (four-and-a-half LIM domains 2), Srf (serum response factor)), apoptosis-related genes (Bax (Bcl-associated X-protein), Bcl2 (Bcl-2), Casp3

(caspase 3)), fibrosis-associated genes (Col1a1, Col3a1 (collagen type III alpha 1 chain), Ctgf (connective tissue growth factor), Fn1 (fibronectin 1), Postn (periostin), S100a4 (S100 Ca^{2+}-binding protein 4)), a pro-inflammatory transcriptional regulator (Nfkb1 (nuclear factor kappa B)) and vascularization markers (Cdh5 (cadherin 5), Vwf (von Willebrand factor); **Figure 6**).

As expected and in line with the qPCR data, the hypertrophic gene program was activated in TAC groups without hHTx/MU. In the 3 and 6 weeks TAC group, expression of hypertrophy marker genes Acta1, Myh7, Nppa, Nppb and the fibroblast activation markers Ctgf, Fn1, Postn and S100a4 was significantly higher and expression of Myh6 was significantly lower than in controls.

Importantly, genes contributing to fibrosis were markedly induced in both hHTx/MU groups. Expression of the fibrosis marker genes Col1a1, Col3a1, Ctgf, Fn1, Postn and S100A4 was higher than in the sham-operated control group, but also compared to the TAC groups. Expression of genes encoding for cardiac myocyte proteins followed no clear pattern. However, in both groups that underwent MU, gene expression of Myh6 was even lower than in the TAC groups and Myh7 gene expression was even higher than after TAC. Nppa and Nppb gene expression was lower than after TAC and almost as low as in sham-operated controls, as expected in response to MU. Furthermore, upregulation of apoptosis-related genes in both hHTx/MU groups could be observed, with gene expression of Bax, Bcl2 and Casp3 higher than in the sham-operated control group and the TAC groups.

The gene expression changes detected by NanoString technology were in accordance with the results obtained by qPCR analysis. In summary, the data confirm activation of fibrosis and apoptosis triggered by hHTx/MU, which occurs regardless of the disease state of the donor heart.

Western immunoblot analysis

It had been reported previously (15) that MU of healthy rat hearts leads to significantly reduced phosphorylation of proteins involved in excitation-contraction coupling and at the same time to reduced total protein levels of sarcomere-associated proteins such as cardiac myosin-binding protein C (cMyBP-C), cardiac troponin I (cTnI) and phospholamban (PLN). Thus, we investigated whether pressure-overload induced hypo-phosphorylation of cardiac proteins - which had been described to associate with contractile dysfunction (22) - recovers after MU. We assessed protein expression and phosphorylation levels of the thick-filament associated protein cMyBP-C, the sarcoplasmic reticulum-associated protein PLN and the thin-filament associated proteins cTnI and tropomyosin 1 (TM1). Additionally, the expression and phosphorylation levels of extracellular-signal-regulated MAPK ERK1/2 were investigated, as they are known contributors to hypertrophic cardiac remodeling. Expression levels of the biomechanical stress sensing transcriptional regulators four-and-a-half-LIM domains isoform 1 (FHL1) and 2 (FHL2) were also investigated, as they are known to impact on the cellular compartmentalization of ERK (23). In hearts subjected to TAC surgery, protein expression of the sarcomeric proteins cMyBP-C, PLN, cTnI and TM1 remained unchanged when compared to sham-operated controls (**Figure 7A-D**). Phosphorylation of cMyBP-C at Ser282, PLN at Ser16 and cTnI at Ser22/23 all typically targeted by cAMP-dependent protein kinase (PKA), was lower in response to pressure overload. In contrast, TM1 phosphorylation at Ser283, most likely phosphorylated by death-associated protein kinase (DAPK, (24), remained unchanged. In samples after hHTx/MU, protein and phosphorylation levels of all sarcomeric proteins tested here were significantly reduced. Even though a decline in total TM1 level was detectable, the phosphorylation of TM1 at Ser283 (**Figure 7D**) was largely preserved compared to the phosphorylation of cMyBP-C, PLN and cTnI (**Figure 7A-C**). ERK MAPK protein levels were not affected by hHTx/MU. Disease progression in response to pressure overload resulted in reduced MEK1-dependent phosphorylation of the canonical ERK phosphorylation sites at Thr202 and Tyr204 (**Figure 7E**; in the rat sequence Thr203, Tyr205 for ERK1 and Thr183, Tyr185 for ERK2), and as expected, in increased autophosphorylation of ERK at Thr188 (**Figure 7F**; in the rat sequence Thr208 in ERK1 and

Thr188 in ERK2), commonly associated with ERK nuclear accumulation and subsequently pro-hypertrophic growth (25). Pan ERK phosphorylation was reduced in samples subjected to hHTx/MU (**Figure 7E,F**). FHL1 protein expression was increased in TAC samples (**Figure 7G**) and FHL2 expression decreased (**Figure 7H**). Protein expression of both proteins remained low after hHTx/MU (**Figure 7G,H**).

Discussion

Commonly, MU by continuous-flow LVAD therapy is considered to be associated with reverse pathological remodeling resulting in a certain degree of cardiac recovery, in turn leading to the possibility of LVAD explantation in approximately 5% of patients. However, it has been demonstrated that myocardial recovery more frequently occurs solely on a molecular and cellular level and without leading to cardiac functional improvements allowing for LVAD removal (26). Recent findings in 37 human specimens suggested regression of cardiac myocyte hypertrophy and improvements in biventricular functional parameters under MU achieved with continuous centrifugal flow LVADs without resulting in transcriptional changes (27). These controversial findings are largely derived from studies performed in non-standardized human specimens with varying disease patterns/states, MU duration and medical regimens and investigations undertaken in mechanical unloaded healthy animal hearts (28).

Here, we performed a systematic molecular analysis of tissue structure, cardiac myocyte size, gene expression and protein expression/phosphorylation pattern in 14-days mechanically unloaded standardized pressure overloaded rat hearts. Main findings were (I) fibrosis development in response to MU independent of disease state or functional parameters prior to hHTx/MU as evidenced by independent methodologies. (II) Regression of cardiac myocyte diameter to sham-operated control levels in response to MU in TAC operated hearts. (III) Reduction in sarcomeric protein content with reduced phosphorylation of common PKA-substrates, but increased phosphorylation of TM1, a substrate for protein kinase C (PKC) and DAPK-mediated phosphorylation. This potentially predicts impaired cardiac contractile performance, enhanced oxidative stress and apoptosis after MU in both, sham-operated and TAC hearts.

Prior investigation of MU in a genetic model of dilated cardiomyopathy showed an association of prolonged LV unloading with impaired myocardial relaxation induced by myocardial atrophy, fibrosis development and apoptosis (18). Similar effects of MU on the

extracellular matrix of the LV were demonstrated for human hearts subjected to LVAD therapy (29). In contrast, other reports showed a significant reduction of LV collagen content after LVAD therapy (30). The experiments conducted in this study provide indices for the activation of the fibrotic remodeling program using histology, assessment of collagen content and gene expression analysis. The development of fibrosis occurred regardless of the disease status of the donor heart. This was not only evident in the PSR-stained cardiac sections and by colorimetric collagen assay, but was further corroborated by qPCR and NanoString technology showing elevated transcription of fibrosis marker genes Col1a1, Col3a1, Ctgf, Fn1, Postn and S100A4 alongside with up-regulation of apoptosis-related genes. Cardiac myocyte diameter was reduced in response to MU, as had been described before (31). Despite the fact that assessment of cardiac function in hearts after hHTx/MU in our study failed, the possibility of cardiac functional recovery seemed unlikely, given the apparent pathological changes in extracellular matrix content, atrophy and apoptosis. However, this remains speculative and needs to be investigated in future studies by directly assessing cardiac function after mechanical unlading by e.g. echocardiography or pressure-volume relationship measurements.

Impairment of contractile function could furthermore be predicted from the results obtained by western immunoblot analysis. In TAC hearts, PKA-mediated phosphorylation of sarcomeric proteins was reduced. This is commonly associated with compromised cardiac performance and reflects the desensitization of the β-adrenoceptor signaling pathway during cardiac disease development due to a deficiency of the cardiac myocytes to adjust contractile function adequately to acute changes in neurohumoral stimulation (as reviewed by Eschenhagen (32)). In addition to the hypo-phosphorylation of sarcomeric proteins in TAC hearts, subsequent hHTx/MU led to a dramatic reduction in sarcomeric protein content, which had been described before (15). A loss of sarcomeric structure, enhanced fibrosis and apoptosis all contribute to impairment of cardiac myocyte contractile function. In addition to our observations regarding structural remodeling, our study for the first time shows that a

reduction in sarcomeric protein content and phosphorylation occurred irrespective of the health condition of the donor heart and can thus be regarded as a generic effect of the unloading procedure.

The impact of oxidative stress on sarcomere function during cardiac disease development has been described before (reviewed by Steinberg (33)). Phosphorylation of the sarcomeric protein TM1 at Ser283 by PKC and DAPK occurs downstream of oxidant-mediated ERK1/2 activation (24) and thus functions as a redox-sensor. In the heart, TM1 phosphorylation has been described to modulate the development of cardiac hypertrophy (34), with high phosphorylation levels of TM1 at Ser283 observed in a mouse model mimicking human hypertrophic cardiomyopathy (35). However, thus far, neither changes in TM1 isoform expression nor in its phosphorylation state could be confirmed in ventricular tissue from patients with end-stage heart failure (36). Our results are in accordance with the literature and show that TM1 expression and phosphorylation levels remain unchanged in hearts in response to pressure overload. However, TM1 protein levels declined in response to hHTx/MU, with the remaining TM1 protein highly phosphorylated at Ser283. This suggests increased generation of reactive oxygen species with subsequent activation of DAPK during TAC and TAC/hHTx/MU. Oxidative stress might also contribute to the induction of the apoptosis-related gene program as evidenced by higher mRNA abundance of Casp3, Bcl2 and Bax compared to sham-operated controls and after TAC surgery.

To investigate the contribution of the Raf-MEK-ERK1/2 pathway to the observed pathological hypertrophic remodeling, ERK1/2 protein expression and phosphorylation levels were assessed. The canonical MEK1/2-dependent ERK1/2 phosphorylation was reduced after TAC surgery. This was paralleled by elevated autophosphorylation of ERK1/2 at Thr188. Autophosphorylation of ERK1/2 is considered to favor nuclear translocation and accumulation of the kinase with subsequent induction of pro-hypertrophic gene expression (25). After hHTx/MU, ERK1/2 autophosphorylation was barely detectable. This observation

can potentially explain the reduction in cardiac myocyte diameter, which we found regressed to sham-operated levels after TAC/hHTx/MU.

Cellular localization of ERK1/2 is regulated in part by the biomechanical stress regulators FHL1 and FHL2. FHL1 has been shown to participate in the formation of a signaling complex with ERK2 that senses hypertrophic stress signals, thereby negatively regulating ERK2-mediated titin phosphorylation and reducing muscle compliance (37). FHL2 has been described to regulate Raf-MEK-ERK1/2 signaling by directly interacting with ERK1/2 and maintaining its cytosolic localization (23). FHL2 protein levels have been described to decrease in human heart failure (38, 39), allowing ERK1/2 nuclear translocation, nuclear accumulation and pro-hypertrophic gene expression. Our study demonstrates increased FHL1 and reduced FHL2 expression after TAC surgery, which is in accordance with elevated ERK1/2 autophosphorylation and cardiac myocyte hypertrophy. In response to hHTx/MU, FHL1 protein levels declined, whilst FHL2 protein levels remained low. This could on the one hand improve muscle compliance and thus cardiac function, but also further aggravate pathological remodeling.

In summary, hHTx/MU of rat hearts revealed exacerbated fibrosis development, cardiac myocyte atrophy and transcription of apoptosis-related genes regardless of the disease condition of the donor heart at the time of transplantation. Whether a step-wise unloading procedure or even an anti-fibrotic therapy would induce superior recovery of the molecular signaling pathways warrants further investigation. The molecular mechanisms that occur in diseased hearts in response to hHTx/MU are summarized in **Figure 8**. Our data in many aspects fit to the clinical situation with low rate of recovery-related LVAD explantation in patients and partially elucidate the molecular remodeling mechanisms.

Study limitations

Main limitation of this study is the small number of biological replicates in the hHTx/MU group. This is due to the sophisticated surgical animal model used in our study in order to address a clinically important question. However, conclusions are drawn based on robust evidence achieved with multiple alternative methodologies. Furthermore, only complete unloading of the LV was performed without comparing effects of partial unloading by heterotopic heart-lung transplantation, which could at least attenuate cardiac fibrosis, atrophy and wall stiffness induced by MU. Moreover, the here described and characterized surgical model only partially represents the clinical situation of LVAD therapy in patients with end-stage heart failure. Important differences to be emphasized are the complete unloading of the heart in our hHTx model, together with different anastomoses (PA to inferior vena cava, AA to abdominal aorta vs. apex to AA) and the non-native position of the transplanted heart, which may lead to artificial interactions with the surrounding abdominal tissue.

Acknowledgments

We thank Kristin Hartmann of the Mouse Pathology Core facility, University Medical Center Hamburg-Eppendorf for generating cardiac tissue sections and histological staining.

Sources of Funding

FC, JS, TE were supported by the DZHK (German Centre for Cardiovascular Research) and the German Ministry of Research and Education (BMBF). This study was supported by a grant from the Deutsche Forschungsgemeinschaft (DFG grant CU 53/2-1; CU 53/5-1) and the Werner-Otto-Stiftung (8/89; 7/92). KL was supported by the German Ministry of Research and Education (BMBF; Comprehensive Heart Failure Center Würzburg) and by the Ministry for Innovation, Science and Research of the Federal State of North Rhine Westphalia.

Disclosure statement/Competing interests: No author of this work has any conflict of interest or competing interest regarding this work.

Data availability statement: The datasets generated during and/or analysed during the current study are available from the corresponding author on reasonable request.

Author contributions statement:

AS developed study/study design, conducted animal experiments, wrote and revised the manuscript

YS developed study/study design, conducted animal experiments, wrote and revised the manuscript

SS conducted western-immunoblotting, revised the manuscript for intellectual content

SK conducted histology, revised the manuscript for intellectual content

TW conducted western-immunoblotting, revised the manuscript for intellectual content

AP conducted western-immunoblotting, revised the manuscript for intellectual content

GH conducted western-immunoblotting, revised the manuscript for intellectual content

CM conducted histology, revised the manuscript for intellectual content

KL provided antibodies, conducted western-immunoblotting, revised the manuscript for intellectual content

DW provided antibodies, conducted western-immunoblotting, revised the manuscript for intellectual content

APS revised the manuscript for intellectual content

TE revised the manuscript for intellectual content

HE revised the manuscript for intellectual content

HR revised the manuscript for intellectual content

JS conducted western-immunoblotting, gene expression analysis, wrote and revised the manuscript

FC conducted western-immunoblotting, gene expression analysis, wrote and revised the manuscript

References

1. Kirklin JK, Naftel DC, Pagani FD, Kormos RL, Stevenson LW, Blume ED, Myers SL, Miller MA, Baldwin JT, Young JB. Seventh INTERMACS annual report: 15,000 patients and counting. J Heart Lung Transplant. 2015 Dec;34(12):1495-504. doi: 10.1016/j.healun.2015.10.003.
2. Kirklin JK, Naftel DC, Pagani FD, Kormos RL, Stevenson LW, Blume ED, Miller MA, Baldwin JT, Young JB. Sixth INTERMACS annual report: a 10,000-patient database. J Heart Lung Transplant. 2014 Jun;33(6):555-64. doi: 10.1016/j.healun.2014.04.010.
3. Gustafsson F, Rogers JG. Left ventricular assist device therapy in advanced heart failure: patient selection and outcomes. Eur J Heart Fail. 2017 May;19(5):595-602. doi: 10.1002/ejhf.779.
4. Braunwald E. The war against heart failure: the Lancet lecture. Lancet. 2015 Feb 28;385(9970):812-24. doi: 10.1016/S0140-6736(14)61889-4.
5. Drakos SG, Mehra MR. Clinical myocardial recovery during long-term mechanical support in advanced heart failure: Insights into moving the field forward. J Heart Lung Transplant. 2016 Apr;35(4):413-20. doi: 10.1016/j.healun.2016.01.001.
6. Frazier OH, Baldwin AC, Demirozu ZT, Segura AM, Hernandez R, Taegtmeyer H, Mallidi H, Cohn WE. Ventricular reconditioning and pump explantation in patients supported by continuous-flow left ventricular assist devices. J Heart Lung Transplant. 2015 Jun;34(6):766-72. doi: 10.1016/j.healun.2014.09.015.
7. Dipla K, Mattiello JA, Jeevanandam V, Houser SR, Margulies KB. Myocyte recovery after mechanical circulatory support in humans with end-stage heart failure. Circulation. 1998 Jun 16;97(23):2316-22.
8. Birks EJ. Molecular changes after left ventricular assist device support for heart failure. Circ Res. 2013 Aug 30;113(6):777-91. doi: 10.1161/CIRCRESAHA.113.301413.
9. Liem DA, Nsair A, Setty SP, Cadeiras M, Wang D, Maclellan R, Lotz C, Lin AJ, Tabaraki J, Li H, Ge J, Odeberg J, Ponten F, Larson E, Mulder J, Lundberg E, Weiss JN, Uhlen M, Ping P, Deng MC. Molecular- and organelle-based predictive paradigm underlying recovery by left ventricular assist device support. Circ Heart Fail. 2014 Mar 1;7(2):359-66. doi: 10.1161/CIRCHEARTFAILURE.113.000250.
10. Mann DL, Barger PM, Burkhoff D. Myocardial recovery and the failing heart: myth, magic, or molecular target? J Am Coll Cardiol. 2012 Dec 18;60(24):2465-72. doi: 10.1016/j.jacc.2012.06.062.
11. Razeghi P, Myers TJ, Frazier OH, Taegtmeyer H. Reverse remodeling of the failing human heart with mechanical unloading. Emerging concepts and unanswered questions. Cardiology. 2002;98(4):167-74.
12. Sakamuri SS, Takawale A, Basu R, Fedak PW, Freed D, Sergi C, Oudit GY, Kassiri Z. Differential impact of mechanical unloading on structural and nonstructural components of the extracellular matrix in advanced human heart failure. Transl Res. 2016 Jun;172:30-44. doi: 10.1016/j.trsl.2016.02.006.
13. Liu Y, Maureira P, Gauchotte G, Falanga A, Marie V, Olivier A, Groubatch F, Gu C, Marie PY, Tran N. Effect of chronic left ventricular unloading on myocardial remodeling: Multimodal assessment of two heterotopic heart transplantation techniques. J Heart Lung Transplant. 2015 Apr;34(4):594-603. doi: 10.1016/j.healun.2014.11.015.
14. Ono K, Lindsey ES. Improved technique of heart transplantation in rats. J Thorac Cardiovasc Surg. 1969 Feb;57(2):225-9.
15. Schwoerer AP, Neuber C, Schmechel A, Melnychenko I, Mearini G, Boknik P, Kirchhefer U, Schmitz W, Ehmke H, Eschenhagen T, El-Armouche A. Mechanical unloading of the rat heart involves marked changes in the protein kinase-phosphatase balance. J Mol Cell Cardiol. 2008 Dec;45(6):846-52. doi: 10.1016/j.yjmcc.2008.09.003.
16. Schwoerer AP, Melnychenko I, Goltz D, Hedinger N, Broichhausen I, El-Armouche A, Eschenhagen T, Volk T, Ehmke H. Unloaded rat hearts in vivo express a hypertrophic phenotype of cardiac repolarization. J Mol Cell Cardiol. 2008 Nov;45(5):633-41. doi: 10.1016/j.yjmcc.2008.02.271.
17. Schaefer A, Schneeberger Y, Stenzig J, Biermann D, Jelinek M, Reichenspurner H, Eschenhagen T, Ehmke H, Schwoerer AP. A New Animal Model for Investigation of Mechanical

Unloading in Hypertrophic and Failing Hearts: Combination of Transverse Aortic Constriction and Heterotopic Heart Transplantation. PLoS One. 2016 Feb 3;11(2):e0148259. doi: 10.1371/journal.pone.0148259.
18. Muranaka H, Marui A, Tsukashita M, Wang J, Nakano J, Ikeda T, Sakata R. Prolonged mechanical unloading preserves myocardial contractility but impairs relaxation in rat heart of dilated cardiomyopathy accompanied by myocardial stiffness and apoptosis. J Thorac Cardiovasc Surg. 2010 Oct;140(4):916-22. doi: 10.1016/j.jtcvs.2010.02.006.
19. McGowan BS, Scott CB, Mu A, McCormick RJ, Thomas DP, Margulies KB. Unloading-induced remodeling in the normal and hypertrophic left ventricle. Am J Physiol Heart Circ Physiol. 2003 Jun;284(6):H2061-8.
20. Schneider CA, Rasband WS, Eliceiri KW. NIH Image to ImageJ: 25 years of image analysis. Nat Methods. 2012 Jul;9(7):671-5.
21. Cuello F, Bardswell SC, Haworth RS, Yin X, Lutz S, Wieland T, Mayr M, Kentish JC, Avkiran M. Protein kinase D selectively targets cardiac troponin I and regulates myofilament Ca2+ sensitivity in ventricular myocytes. Circ Res. 2007 Mar 30;100(6):864-73.
22. El-Armouche A, Pohlmann L, Schlossarek S, Starbatty J, Yeh YH, Nattel S, Dobrev D, Eschenhagen T, Carrier L. Decreased phosphorylation levels of cardiac myosin-binding protein-C in human and experimental heart failure. J Mol Cell Cardiol. 2007 Aug;43(2):223-9.
23. Purcell NH, Darwis D, Bueno OF, Muller JM, Schule R, Molkentin JD. Extracellular signal-regulated kinase 2 interacts with and is negatively regulated by the LIM-only protein FHL2 in cardiomyocytes. Mol Cell Biol. 2004 Feb;24(3):1081-95.
24. Houle F, Poirier A, Dumaresq J, Huot J. DAP kinase mediates the phosphorylation of tropomyosin-1 downstream of the ERK pathway, which regulates the formation of stress fibers in response to oxidative stress. J Cell Sci. 2007 Oct 15;120(Pt 20):3666-77.
25. Lorenz K, Schmitt JP, Schmitteckert EM, Lohse MJ. A new type of ERK1/2 autophosphorylation causes cardiac hypertrophy. Nat Med. 2009 Jan;15(1):75-83. doi: 10.1038/nm.1893.
26. Rossing K, Gustafsson F. Medical and mechanical unloading in advanced heart failure: hope for cardiac recovery? Eur J Heart Fail. 2018 Jan;20(1):175-177. doi: 10.1002/ejhf.1081.
27. Muthiah K, Humphreys DT, Robson D, Dhital K, Spratt P, Jansz P, Macdonald PS, Hayward CS. Longitudinal structural, functional, and cellular myocardial alterations with chronic centrifugal continuous-flow left ventricular assist device support. J Heart Lung Transplant. 2017 Jul;36(7):722-731. doi: 10.1016/j.healun.2016.05.017.
28. Bruckner BA, Razeghi P, Stetson S, Thompson L, Lafuente J, Entman M, Loebe M, Noon G, Taegtmeyer H, Frazier OH, Youker K. Degree of cardiac fibrosis and hypertrophy at time of implantation predicts myocardial improvement during left ventricular assist device support. J Heart Lung Transplant. 2004 Jan;23(1):36-42.
29. Klotz S, Foronjy RF, Dickstein ML, Gu A, Garrelds IM, Danser AH, Oz MC, D'Armiento J, Burkhoff D. Mechanical unloading during left ventricular assist device support increases left ventricular collagen cross-linking and myocardial stiffness. Circulation. 2005 Jul 19;112(3):364-74.
30. Maybaum S, Mancini D, Xydas S, Starling RC, Aaronson K, Pagani FD, Miller LW, Margulies K, McRee S, Frazier OH, Torre-Amione G; LVAD Working Group. Cardiac improvement during mechanical circulatory support: a prospective multicenter study of the LVAD Working Group. Circulation. 2007 May 15;115(19):2497-505.
31. Brinks H, Tevaearai H, Mühlfeld C, Bertschi D, Gahl B, Carrel T, Giraud MN. Contractile function is preserved in unloaded hearts despite atrophic remodeling. J Thorac Cardiovasc Surg. 2009 Mar;137(3):742-6. doi: 10.1016/j.jtcvs.2008.09.020.
32. Eschenhagen T. Beta-adrenergic signaling in heart failure-adapt or die. Nat Med. 2008 May;14(5):485-7. doi: 10.1038/nm0508-485.
33. Steinberg SF. Oxidative stress and sarcomeric proteins. Circ Res. 2013 Jan 18;112(2):393-405. doi: 10.1161/CIRCRESAHA.111.300496.
34. Schulz EM, Correll RN, Sheikh HN, Lofrano-Alves MS, Engel PL, Newman G, Schultz Jel J, Molkentin JD, Wolska BM, Solaro RJ, Wieczorek DF. Tropomyosin dephosphorylation results in

compensated cardiac hypertrophy. J Biol Chem. 2012 Dec 28;287(53):44478-89. doi: 10.1074/jbc.M112.402040.
35. Schulz EM, Wilder T, Chowdhury SA, Sheikh HN, Wolska BM, Solaro RJ, Wieczorek DF. Decreasing tropomyosin phosphorylation rescues tropomyosin-induced familial hypertrophic cardiomyopathy. J Biol Chem. 2013 Oct 4;288(40):28925-35. doi: 10.1074/jbc.M113.466466.
36. Marston SB, Copeland O, Messer AE, MacNamara E, Nowak K, Zampronio CG, Ward DG. Tropomyosin isoform expression and phosphorylation in the human heart in health and disease. J Muscle Res Cell Motil. 2013 Aug;34(3-4):189-97. doi: 10.1007/s10974-013-9347-8.
37. Raskin A, Lange S, Banares K, Lyon RC, Zieseniss A, Lee LK, Yamazaki KG, Granzier HL, Gregorio CC, McCulloch AD, Omens JH, Sheikh F. A novel mechanism involving four-and-a-half LIM domain protein-1 and extracellular signal-regulated kinase-2 regulates titin phosphorylation and mechanics. J Biol Chem. 2012 Aug 24;287(35):29273-84. doi: 10.1074/jbc.M112.372839.
38. Bovill E, Westaby S, Crisp A, Jacobs S, Shaw T. Reduction of four-and-a-half LIM-protein 2 expression occurs in human left ventricular failure and leads to altered localization and reduced activity of metabolic enzymes. J Thorac Cardiovasc Surg. 2009 Apr;137(4):853-61. doi: 10.1016/j.jtcvs.2008.09.006.
39. Okamoto R, Li Y, Noma K, Hiroi Y, Liu PY, Taniguchi M, Ito M, Liao JK. FHL2 prevents cardiac hypertrophy in mice with cardiac-specific deletion of ROCK2. FASEB J. 2013 Apr;27(4):1439-49. doi: 10.1096/fj.12-217018.

Figure 1 - Group design

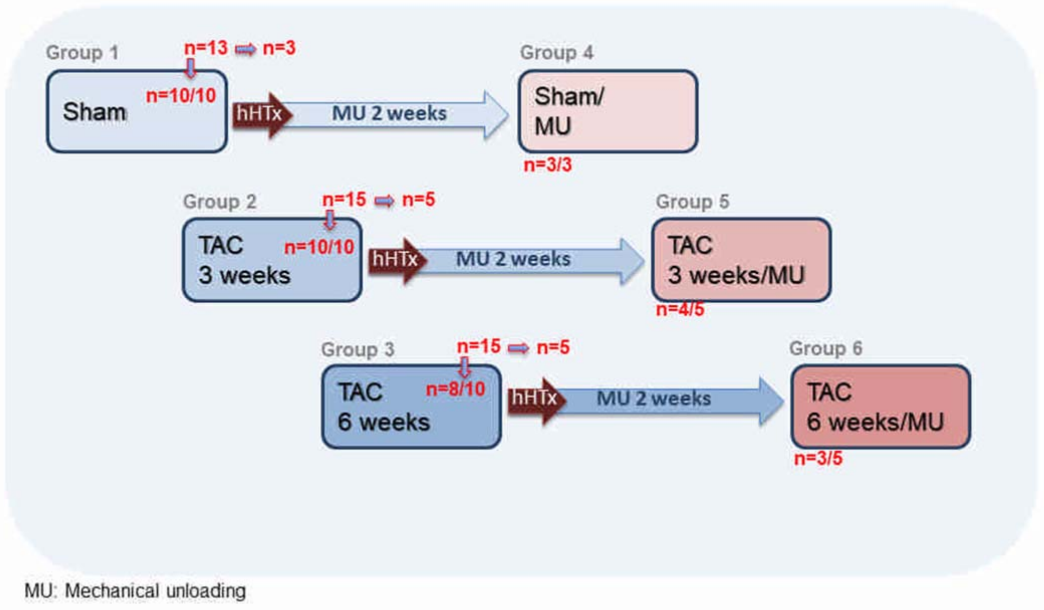

Schematic overview of the study groups with the sham-operated control group (group 1; n=10), the groups of animals that underwent TAC surgery for 3 weeks (group 2; n=10) or 6 weeks (group 3; n=8) and the groups of animals that underwent subsequent heterotopic heart transplantation with mechanical unloading (MU) for additional 2 weeks after sham operation (group 4; n=3), after TAC for 3 weeks (group 5; n=4) and TAC for 6 weeks (group 6; n=3).

Figure 2- Histological analyses of sham and TAC hearts

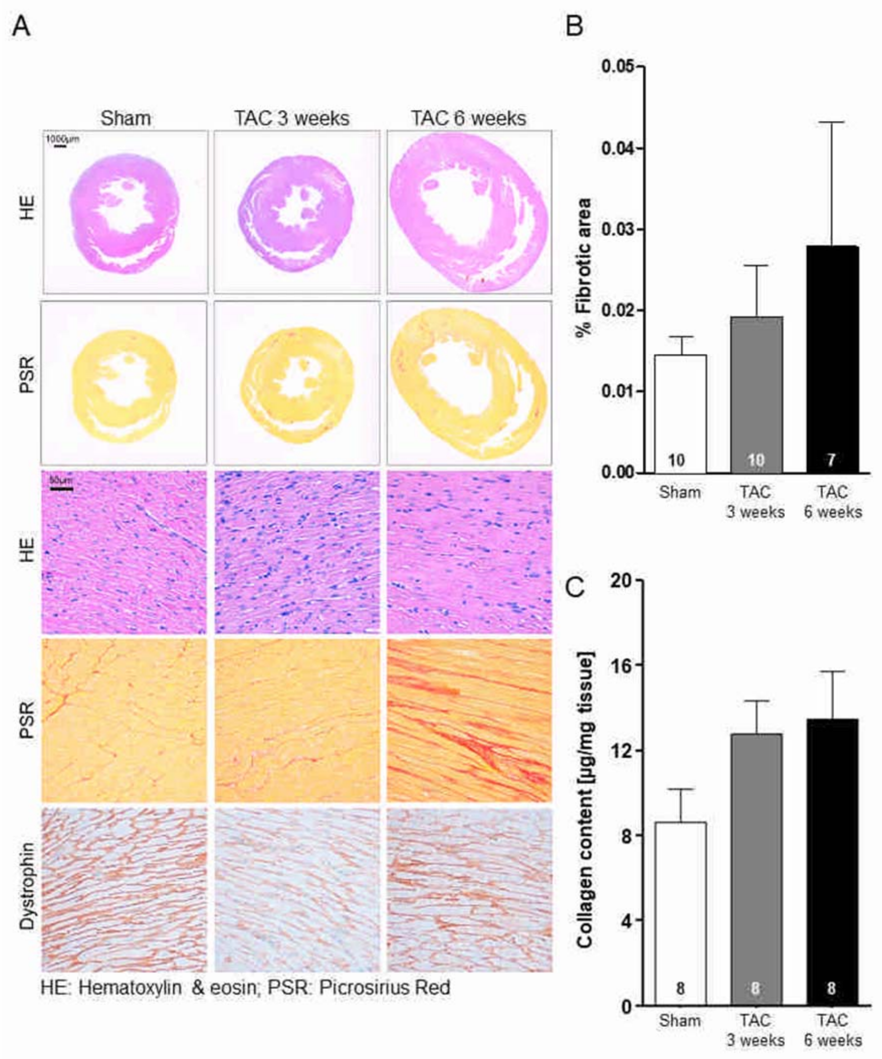

Hearts were removed after sham-operation (n=10) or 3 (n=10) or 6 (n=7) weeks after TAC surgery, paraffin-embedded and transverse cardiac sections were generated. Sections were subjected to hematoxylin/eosin (H&E), Picrosirius Red (PSR) or dystrophin staining. **A)** Representative sections. **B)** Quantification of fibrotic area (%) from PSR staining of 7-10 hearts per group. **C)** Quantification of total collagen content by colorimetric assay (n=8 per group). One-way ANOVA followed by Dunnett's post-test (to sham). Bars display mean±SEM. No significant differences.

Figure 3 - Histological analysis of sham and TAC hearts after hHTx/MU

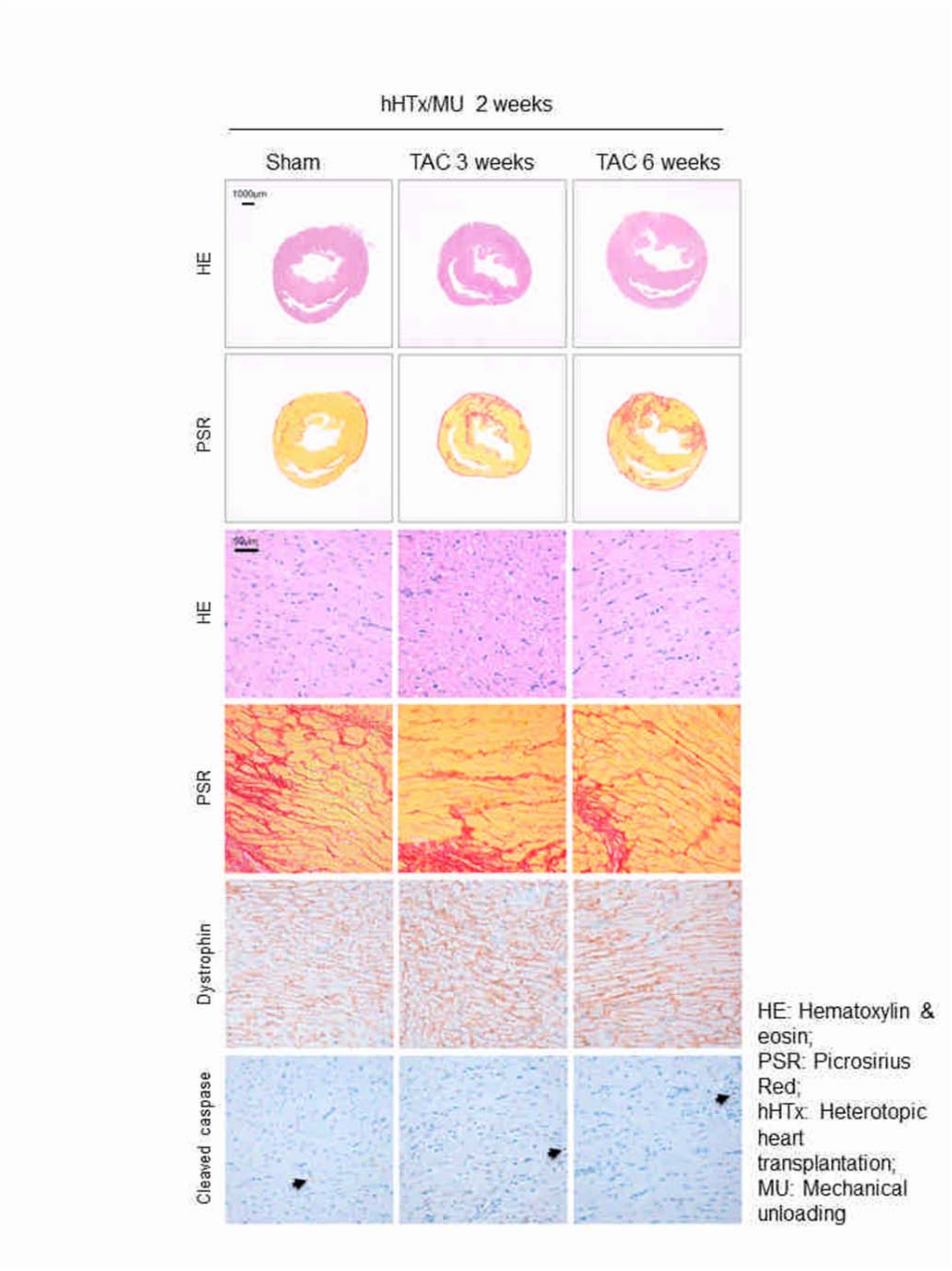

Hearts after sham-operation (n=3) or 3 (n=4) or 6 (n=3) weeks after TAC surgery underwent heterotopic heart transplantation with mechanical unloading for 2 weeks (hHTx/MU). Hearts were paraffin-embedded and transverse cardiac sections generated. Sections were

subjected to hematoxylin/eosin (H&E), Picrosirius Red (PSR), dystrophin or caspase staining (arrows pointing at putative apoptotic nuclei).

Figure 4 - Cardiac myocyte diameter and fibrotic remodeling

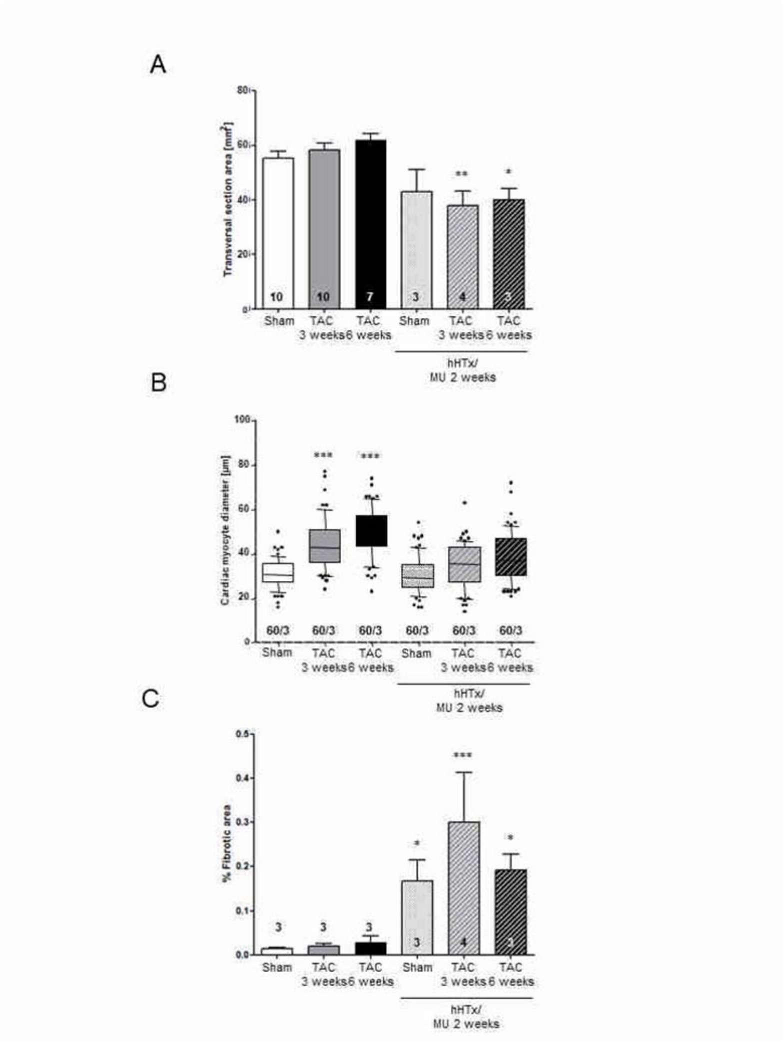

Transverse cardiac sections were stained for fibrosis or dystrophin. **A)** Transverse cardiac section area in mm^2 directly below the mitral valve was assessed in sham-operated (n=10) hearts or after TAC surgery for 3 (n=10) or 6 (n=7) weeks or after hHTx/MU (n=3-4). Quantification was performed from images from Figure 2 and 3. **B)** Cardiac myocyte diameter in µm was assessed after dystrophin staining (20 cells each from 3 animals per group). Significance tested vs. sham using Generalized Estimation Equation for clustered data. ***P<0.001. Boxes display mean±SEM, whiskers display 10 to 90 percentile range. Dots represent outliers which were included in the statistical analysis. **C)** Fibrotic area in %

was assessed after PSR staining. One-way ANOVA followed by Dunnett's post-test (to sham). Bars display mean±SEM. *P<0.05; **P<0.005; *** P<0.001

Figure 5 - Gene expression analysis by qPCR

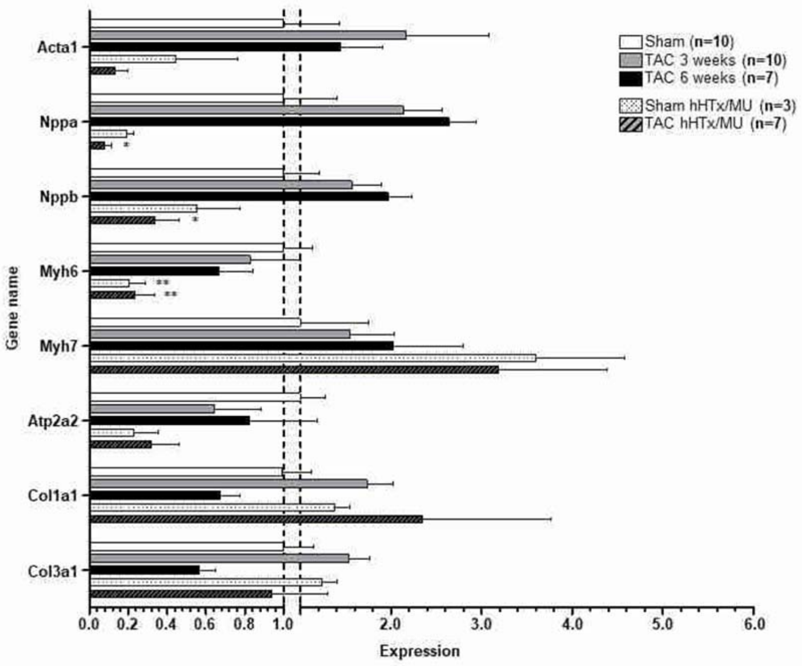

Expression of Acta1, Nppa, Nppb, Myh6, Myh7, Atp2a2, Col1a1, and Col3a1 was assessed by quantitative PCR. Due to the small number of samples available in TAC group 3 and 6 weeks after hHTx/MU, the results were pooled and the resulting group was labeled TAC/hHTx/MU. One-way ANOVA followed by Dunnett's post-test (to sham). Bars display mean±SEM. *P<0.05; **P<0.005; Acta1: alpha 1 skeletal muscle actin; Nppa: natriuretic peptide A; Nppb: natriuretic peptide B; Myh6: alpha-myosin heavy chain; Myh7: beta-myosin

heavy chain; Atp2a2: sarcoplasmic/endoplasmic reticulum Ca^{2+}-ATPase 2; Col1a1: collagen type I alpha 1 chain; Col3a1: collagen type I alpha 3 chain

Figure 6 - Gene expression analysis by NanoString technology

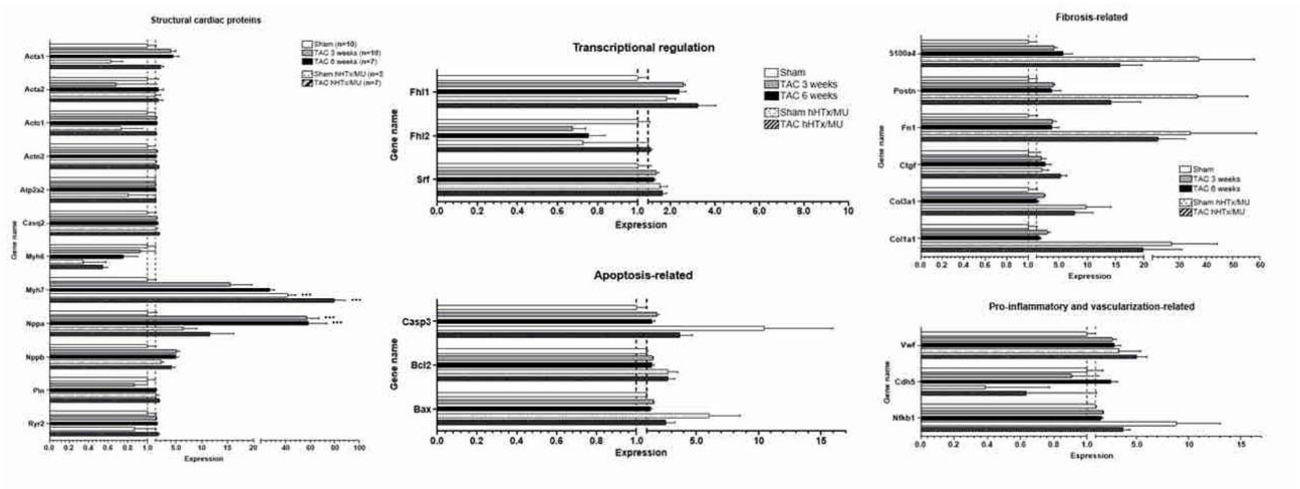

Expression of a panel of 27 genes was assessed by NanoString technology. Genes were grouped according to the function of the encoded protein into structural cardiac proteins, transcriptional regulators, apoptosis-related, fibrosis-related, pro-inflammatory and vascularization-related. One-way ANOVA followed by Dunnett's post-test (to sham) for each gene and subsequent adjustment for multiple testing using the Benjamini-Hochberg procedure. Bars display mean±SEM. ***P*<0.005*;* *** *P*<0.001; Acta1: alpha 1 skeletal muscle actin; Acta2: alpha 2 skeletal muscle actin; Actc1: cardiac muscle alpha actin; Actn2: alpha actinin 2; Atp2a2: sarcoplasmic/endoplasmic reticulum Ca^{2+}-ATPase 2; Casq2: calsequestrin 2; My6: alpha-myosin heavy chain; My7: beta-myosin heavy chain; Nppa: natriuretic peptide A; Nppb: natriuretic peptide B; Pln: phospholamban; Ryr2: ryanodine receptor 2; Fhl1: four-and-a-half LIM domains 1; Fhl2: four-and-a-half LIM domains 2; Srf: serum response factor; Casp3: caspase 3; Bcl2: Bcl-2; Bax: Bcl-associated X-protein; S100a4: S100 Ca^{2+}-binding protein 4; Postn: periostin; Fn1: fibronectin 1; Ctgf: connective tissue growth factor; Col3a1: collagen type III alpha 1 chain; Col1a1: collagen type 1 alpha 1 chain; Vwf: von Willebrand factor; Cdh5: cadherin 5; Nfkb1: nuclear factor kappa B

Figure 7 - Protein expression and phosphorylation

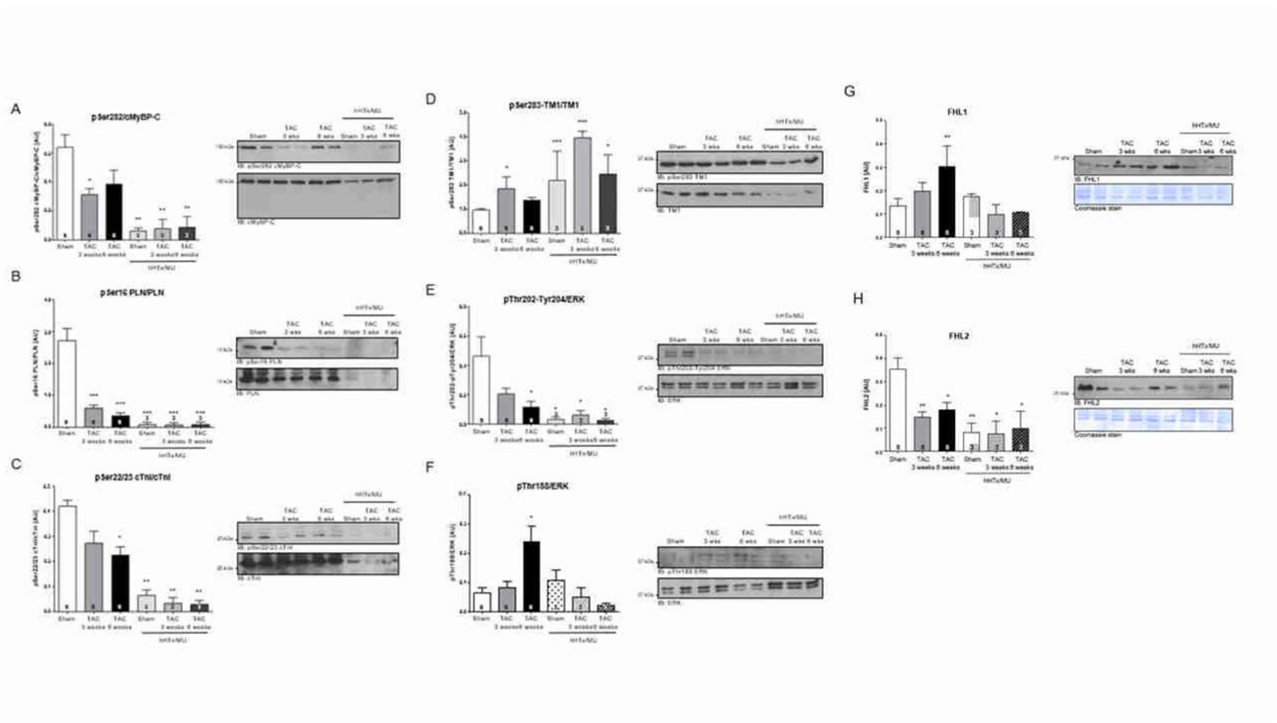

Western immunoblot analysis of cardiac tissue homogenates from sham-operated control rats (sham; n=8), after TAC surgery (TAC, 3 weeks; 6 weeks; n=8) or after hHTx/MU for 14 days (Sham/hHTx/MU; TAC 3 weeks/hHTx/MU; TAC 6 weeks/hHTx/MU; n=3-4). Protein expression and phosphorylation was assessed for **A)** cMyBP-C and pSer282; **B)** PLN and pSer16; **C)** cTnI and pSer22/23; **D)** TM1 and pSer283; **E)** ERK1/2 and pThr202/pTyr204; **F)** ERK1/2 and pThr188; **G)** FHL1; **H)** FHL2. Bars display quantification results as mean±SEM. One-way ANOVA followed by Dunnett's post-test (to sham). **P*<0.05; ***P*<0.005; ****P*< 0.001 (For raw data / full western immunoblots for data shown in figure 7 see **Supplementary figure 1**)

Figure 8 - Summary scheme

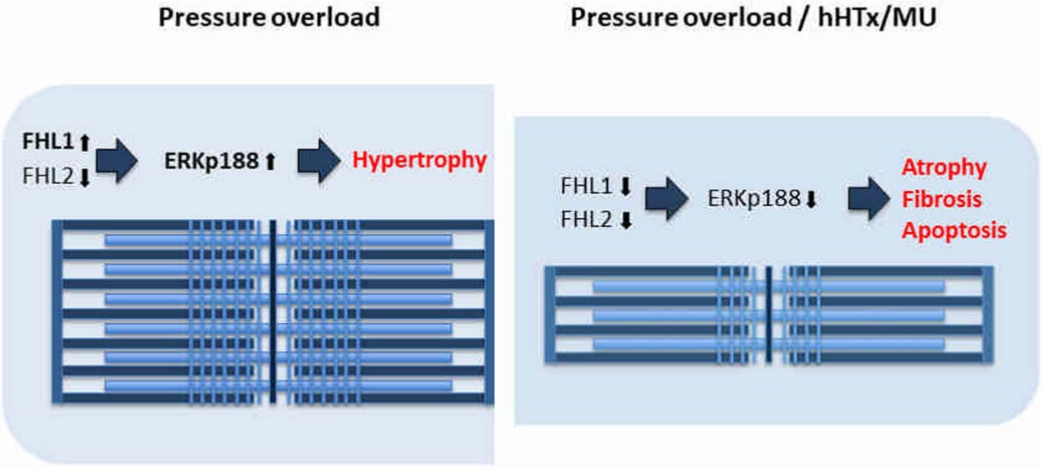

Summary of molecular alterations in cardiac myocytes after TAC or TAC and hHTx/MU

	Sham (n=10)	TAC 3 weeks (n=10)	TAC 6 weeks (n=8)	p-value Sham vs. TAC 3 weeks	p-value Sham vs. TAC 6 weeks	p-value TAC 3 vs. TAC 6 weeks
**TTE 2 weeks after TAC**						
Δp^{max} (mmHg)	/	72.7±22.7	50.9±4.1	/	/	0.02
Δp^{mean} (mmHg)	/	24.4±7.3	17.3±1.5	/	/	0.02
**TTE 3/6 weeks after TAC**						
Ejection fraction (%)	52.1±2.3	33.8±4.5	20.5±16.1	<0.0001	<0.0001	0.02
Fractional shortening (%)	28.2±1.5	23.7±4.3	17.1±3.2	< 0.01	<0.0001	< 0.01
Wall thickness (mm)	1.9±0.1	2.2±0.3	2.1±0.2	< 0.01	0.01	0.43
Left ventricular surface (mm²)	9.6± 2.4	5.8±1.0	19.9±1.1	< 0.001	<0.0001	<0.0001
Enddiastolic volume (µl)	580.1±19.5	1407.7±250.0	1596.4±211.7	<0.0001	<0.0001	0.11
Endsystolic volume (µl)	278.3±18.5	982.6±135.8	1287.9±388.0	<0.0001	<0.0001	0.03
**Clinical phenotype**						
Body Weight (g)	254.7±70.5	185.7±24.1	299.4±45.8	< 0.01	0.14	<0.0001
Heart weight (g)	0.9±0.2	1.1±0.2	1.8±0.3	0.04	<0.0001	<0.0001
HW/BW ratio	0.004±0.0003	0.006±0.0007	0.006±0.002	<0.0001	< 0.01	1.0
Tibia length (mm)	31.7±2.9	28.3±0.5	34.7±0.7	< 0.01	0.01	<0.0001

Table 1 Echocardiography and clinical phenotype after TAC: Summary of the functional and phenotypical data after sham-operation or TAC surgery. *P*-values are given for comparison of sham-operated versus TAC 3 weeks; sham-operated vs TAC 6 weeks; TAC 3 weeks vs TAC 6 weeks. One-way ANOVA followed by Dunnett's post-test (to sham).

Supplemental Table 1

Sequences for the primer pairs used for qPCR based expression analysis

Supplemental Table 2

Raw values of the NanoString analysis

Supplementary Figure 1

Raw data / full western immunoblots for figure 7
